# Increasing tidal inundation corresponds to rising porewater nutrient concentrations in a southeastern U.S. salt marsh

**DOI:** 10.1371/journal.pone.0278215

**Published:** 2022-11-28

**Authors:** Julie L. Krask, Tracy L. Buck, Robert P. Dunn, Erik M. Smith

**Affiliations:** North Inlet-Winyah Bay National Estuarine Research Reserve, Baruch Institute for Coastal and Marine Sciences, University of South Carolina, Georgetown, SC, United States of America; University of South Florida, UNITED STATES

## Abstract

Salt marshes are ecologically and economically important features of coastal environments that are vulnerable to sea level rise, the rate of which has accelerated in recent decades along the southeastern US Atlantic coast. Increased flooding frequency and duration across the marsh platform is predicted to impact vegetation community structure and overall marsh persistence, but the effect of changing inundation patterns on biogeochemical processes in marsh sediments remains largely unexplored. As part of a long-term monitoring effort to assess how marshes are responding to sea level rise in North Inlet estuary (South Carolina, USA), we collected data on porewater nutrient concentrations from a series of permanent monitoring plots across multiple transects spanning the marsh elevation gradient during the growing season from 2009 to 2019. Additionally, we calculated time inundated for each plot using local water level data and high-resolution elevation measurements to assess the change in time flooded at each plot. Our results indicate that both NH_4_ and PO_4_ nutrient concentrations have increased in most permanent plots over the 11-year study period and that nutrient concentrations are higher with increasing proximity to the creek. Spatial patterns in nutrient increases through time are coincident with considerable increases in tidal inundation observed over the marsh platform. Across plots located in the low marsh, porewater NH_4_ and PO_4_ concentrations have risen at average rates of 8.96 μM/year and 0.86 μM/year, respectively, and have reached rates as high as 27.25 μM/year and 3.13 μM/year. We suggest that increased inundation time due to rising sea level has altered biogeochemical conditions influencing nutrient availability in marsh porewater, resulting in increases that likely have relevance for larger scale nutrient cycles as well as marsh ecosystem stability and function.

## Introduction

Tidal salt marshes are transitional features of coastal landscapes that fuel ecological processes in estuaries and provide numerous ecosystem functions and economically valuable services. Specifically, these vegetated wetlands foster high levels of productivity, provide a complex mosaic of habitat types for faunal communities (including commercially harvested species of fish and invertebrates), serve as buffers for upland landscapes against storms and waves, absorb nutrients and pollutants, and sequester disproportionately high quantities of carbon per unit area [1–6]. Along the southeast US Atlantic coast, salt marshes are characterized by wide, gently sloping marsh platforms that link upland forested landscapes to meandering creeks connecting to the ocean [7]. The subtle elevation gradient between the forest edge and the creek bank results in variable tidal inundation patterns across the marsh platform, which, in turn, mediates biotic and abiotic conditions directly relevant to salt marsh function, including plant zonation patterns, species trophic interactions, and sediment biogeochemistry ([8–12]).

Salt marshes are vulnerable to natural disturbances and anthropogenic stressors on multiple spatial and temporal scales. Historically, humans have modified or eliminated marsh ecosystems in local and regional settings via coastal development and land-use changes that alter run-off, affect drainage and sediment supply patterns, increase exposure to pollutants, and inhibit upland transgression [13, 14]. At larger scales, the long-term viability of salt marsh systems is threatened by climate change and the accelerating pace of sea level rise, which is ultimately predicted to drive marsh migration into upland coastal forests and drown landscapes with insufficient vertical accretion rates ([7, 15–18]). In the interim, rising sea level is increasing tidal flooding frequency at higher elevations across the marsh platform and prolonging inundation times at lower elevations.

Given that tidal inundation is an overarching driver of most salt marsh biogeochemical processes, changing flooding patterns are likely to affect the complex cycles of biologically important compounds within salt marsh sediments and across the sediment—surface water interface [19]. During tidal flooding, oxygenated surface water overlays and infiltrates anoxic salt marsh sediments, resulting in steep redox gradients that fuel respiration of organic matter trapped and generated by salt marsh vegetation [20–22]. Rates and pathways of organic matter respiration are further modulated by the cycling of inorganic nutrients within marsh sediments and porewater, which directly govern vegetation growth [23–25]. Furthermore, models suggest that modified tidal inundation patterns can alter groundwater flow paths and flux volumes from the marsh platform to surrounding creeks and that groundwater flushing is greater when the marsh platform becomes inundated at high tide [26, 27]. This is particularly important because enhanced groundwater flux may result in increased nutrient export from the marsh into tidal creeks given that marsh porewaters are highly enriched in nutrients relative to estuarine surface waters [27, 28]. Thus, changing tidal flooding frequency and duration as a function of sea level rise likely affects salt marsh biogeochemical cycling in numerous ways that may have broader ecosystem implications. Nevertheless, specific impacts of sea-level rise on salt marsh biogeochemistry remains largely understudied.

Along the South Carolina coast, relative mean sea level has risen at a rate of 3.39 mm/year over the last century according to historic tide gauge records at Charleston Harbor [29]. However, more recently the rate of increase in mean sea level has approximately quadrupled, reaching 13.2 mm/year during the period of 2009 to 2019 (S1 Fig). Here, we utilize data collected during this recent period of increased sea level rise to investigate whether changes in tidal inundation patterns correspond to spatial and temporal changes in dissolved inorganic nutrients in marsh sediment porewater. These data were collected as part of a long-term monitoring program established by the North Inlet–Winyah Bay National Estuarine Research Reserve to assess and quantify the responses of a southeastern United States salt marsh to sea level rise. We used high-resolution elevation and local water level data to calculate inundation times across a representative, relatively undisturbed salt marsh. We compared rates of change in inundation time with clear directional trends in porewater nutrient concentrations. Despite spatial variability derived from the many biotic and edaphic factors regulating nutrient availability in salt marshes, we demonstrate a strong positive relationship between rates of change in inundation across the marsh platform and rates of change in porewater nutrient availability. These data suggest that the considerable increases in nutrient concentrations observed in salt marsh porewaters are occurring in response to sea level rise.

## Methods

### Site description

The North Inlet-Winyah Bay National Estuarine Research Reserve (NI-WB NERR) is located in Georgetown, South Carolina (USA) and encompasses two connected estuarine systems: North Inlet, which is a small, bar-built, ocean-dominated estuary, and Winyah Bay, which is a large coastal plain, river-dominated estuary. Our focus here is on the North Inlet estuary, which consists of approximately 33 km^2^ of salt marsh dominated by *Spartina alterniflora* and well-mixed tidal creeks and is surrounded by a largely undeveloped watershed [30, 31]. Freshwater input into the North Inlet system is limited to shallow groundwater and surface run-off from the surrounding watershed associated with episodic precipitation events [32] and periodic pulses from adjacent Winyah Bay during periods of high river discharge via connected tidal creeks [33]. Our study took place within the salt marsh platform of Crabhaul Creek, which is located along the western edge of North Inlet estuary in a subbasin bordered by a forested relic dune ridge to the east and upland oak-pine forest to the west [15, 34]. The head of Crabhaul Creek, and its associated marsh platform, is actively expanding South and West, transgressing forest habitat on its western, upland edge. As such, the Creek axis represents a chronosequence of salt marsh development in North Inlet [15]. Net freshwater input to the Crabhaul creek subbasin of North Inlet has been estimated to be 0.9% of the total tidal influx over the year [26].

**Fig 1:**
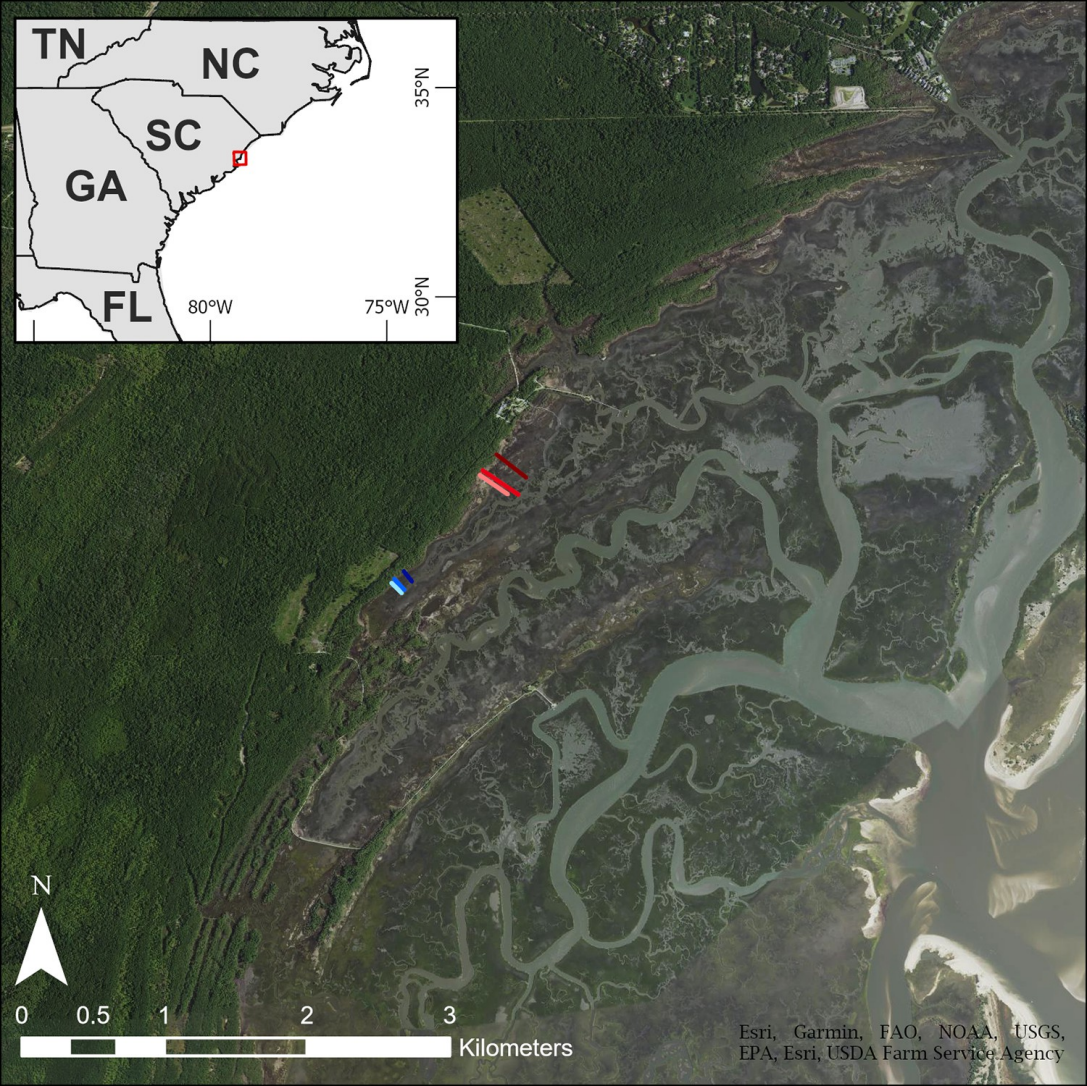
Map of study site. Aerial view of North Inlet Estuary (South Carolina, USA), where the North Inlet–Winyah Bay National Estuarine Research Reserve (NI-WB NERR) has established permanent vegetation monitoring program along the marsh platform of Crabhaul Creek. 51-1m^2^ permanent monitoring plots comprise 6 transects across two distinct segments of the marsh platform. The red lines represent transects at Segment A, while the blue lines represent transects at Segment B. Content is the intellectual property of Esri and is used herein with permission. Copyright © 2022 Esri and its licensors. All rights reserved.

As part of a larger, national NERRS effort, and following established marsh monitoring protocols [35], the NI-WB NERR established 51 1-m^2^ permanent vegetation monitoring plots in a series of six transects divided across two distinct segments of the Crabhaul Creek marsh platform, denoted Segment A and Segment B (Fig 1; S2 Fig). Access to and sampling of this site for long-term research was granted by the Belle W. Baruch Foundation as part of a long-standing partnership between the Foundation, University of South Carolina, and NI-WB NERR, so no specific permit was required. Segments A and B each consists of three parallel transects connecting eight or nine permanent plots which span the marsh elevation gradient, beginning at the forest edge and ending at the creek bank. Segment A represents a geologically older segment of the marsh basin [15] and is approximately 1,000 m downstream from Segment B, which is located near the creek terminus.

The width of the marsh platform (distance from the upland edge of the marsh to the creekbank) is approximately three times greater at Segment A than at Segment B. The wider marsh platform at Segment A coincides with increased diversity with respect to the vegetation community due to the presence of a diverse, mixed species high- and mid-marsh area. This area comprises between half and two thirds of the marsh width and the remaining distance to the creek bank is dominated by monospecific *S*. *alterniflora*. The high diversity mid-marsh includes a mosaic of mixed-meadow species such as *Borrichia frutescens*, *Spartina patens* and *Distichlis spicata*, while the high marsh is dominated by *Juncus roemerianus*. Present also in the mid-marsh at Segment A are areas of barren salt panne surrounded by large areas of the more salt tolerant *Salicornia* species. In contrast, the geologically younger Segment B has a much abbreviated marsh platform where the *S*. *alterniflora* monoculture low marsh covers 95% of the distance from upland to creek, with only a thin 3–5 m strip of high marsh composed primarily of *J*. *roemerianus* and *S*. *patens*. Segment B has no diverse mid-marsh mixed species zone. Both segments are prone to disturbance from wrack deposition, typically along the mid/high marsh border, which leads to frequent shifts in plant community composition as species compete for the newly created open space within these wrack-disturbed areas.

### Porewater sampling

During the growing season (May–August) from 2009 to 2019, we measured NH_4_ and PO_4_ concentrations as well as salinity, in marsh porewater monthly at each of the 51 permanent vegetation monitoring plots. Triplicate porewater samples were collected each month from each plot, with sample locations immediately adjacent to three corners of each permanent plot at a depth of 25 cm using modified porewater diffusion equilibrators [36]. Equilibrators contained 20mL glass scintillation vials filled with deionized water and covered with nitex mesh (20 μm) membranes which were secured with an open-topped screw cap. Vials were placed in porous PVC pipes driven into the marsh sediment where they were left to equilibrate for approximately one month. Upon retrieval, samples were capped and placed on ice in the field. Upon returning to the lab, sample salinities were measured using a Mettler-Toledo Seven2Go pro conductivity probe and samples were then filtered using Whatman 0.45 μm GF/F filters. Filtered samples were analyzed for nutrients within 24 hours using a Seal Analytical AA3 Segmented Flow AutoAnalyzer following standardized colorimetric methods [37–40].

### Elevation and inundation

Elevation profiles of the marsh platform along the six transects and at each permanent plot were determined in 2013 using a Trimble R8 GPS-real-time kinematic system and referenced to the North American Vertical Datum of 1988 (NAVD 88). Elevation measurements were then used in conjunction with water level data from the NOAA tide gauge at Oyster Landing (Station ID: 8662245) to calculate the total number of hours each monitoring plot was inundated per year from 2009 to 2019. This tide station, located at the mouth of Crabhaul Creek is approximately 700 m downstream of Segment A. We used an Excel macro to scan verified 6-minute water level data (datum NAVD 88) downloaded from this station for instances in which tide levels exceeded permanent plot elevation. For these instances, the macro then calculated total inundation frequency and duration at each plot, the total number of hours plots were flooded for each year. We also used the total number hours inundated per year to calculate the percentages of each year that individual plots were flooded.

### Data analysis

To examine spatial patterns in porewater nutrient concentrations across the marsh platform, we fit linear mixed effect models using the nlme package in the R computing environment [41]. We grouped permanent plots into high, mid, and low marsh zones at each segment based on elevation and the dominant vegetation species in each plot in accordance with established classification regimes for vegetation communities in salt marshes (S1 Table) [10, 42]. To assess changes in nutrient concentrations across marsh zones and over time, we calculated the mean porewater nutrient concentration from each plot (based on 3 replicate porewater wells) in each month for both the first and final years of the time series. Nutrient concentrations were log transformed to better meet the model assumptions. We constructed separate models for both nutrient parameters for the two marsh segments because of their distinct geologic histories and because of the differences in zonation and plant community composition as described above. In each model, we included year (2009 or 2019), marsh zone (high, mid, or low) and their interaction as fixed effects. To account for the four replicate measurements collected at each plot during the four months of the growing season in each year, we included a random effect of month nested within year. The effects of the model components on nutrient concentrations were considered significant if *p* <0.05. Additionally, we calculated the marginal and conditional R^2^ for each model using the piecewiseSEM package in R [43].

We used linear regressions to assess plot-level trends in inundation time, porewater nutrient concentrations and porewater salinity over the 11-year period of data collection. For all linear regressions, year was the continuous, independent predictor variable. For the porewater nutrient and salinity models, triplicate measurements collected monthly at each plot were averaged to obtain monthly estimates which we then treated as replicates within each year (n = 44 for each permanent plot). Change in plot inundation time was estimated as the number of hours each plot was inundated per year (n = 11 for each permanent plot). The slopes of the linear fits with *p*-values <0.05 were extracted and presented as rates of change in each parameter at each of the 51 permanent plots.

Finally, we used linear regressions to test for a relationship between the rates of change in porewater nutrient concentrations and rates of change in inundation time across all 51 permanent plots. Slopes from the plot-level linear models of inundation time (in hours inundated per year) were fit against slopes from the models for both porewater NH_4_ and PO_4_ concentrations from 2009–2019.

## Results

**Fig 2:**
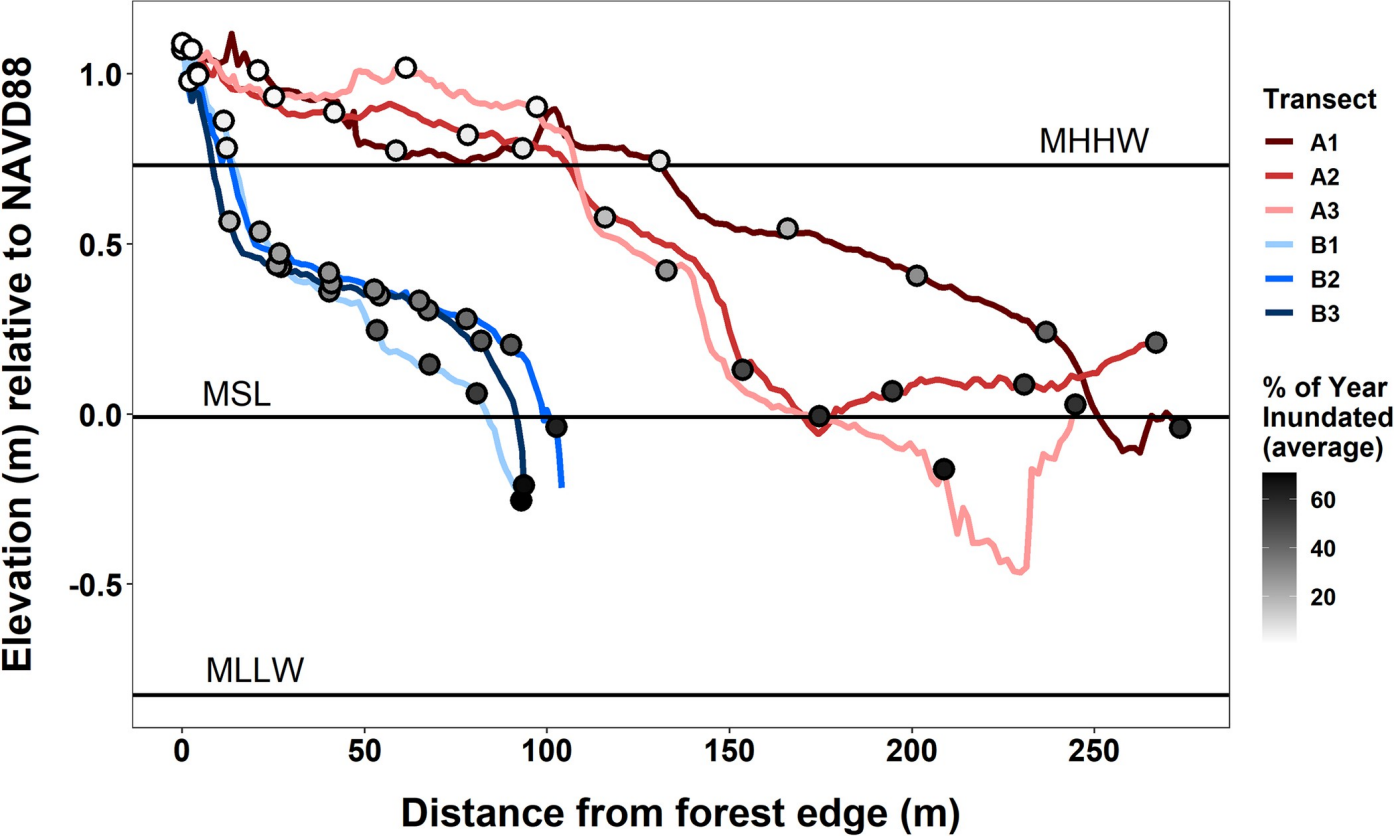
Transect elevation profiles. Elevation profiles estimated using RTK-GPS of the three transects taken across both Segment A (red lines) and Segment B (blue-green lines) of the Crab Haul Creek marsh platform. Points on each profile indicate the location of permanent vegetation monitoring plots designated along each transect and are shaded according to the average of the percentages of each year the plots were inundated for the entire 2009–2019 period.

Transects at Segments A and B differed in distance from the forest edge to the creek bank but covered similar elevation ranges (Fig 2). Transects at Segment A were between 244 and 273 m long and covered an elevation range of -0.467 at the creekbank to 1.09 m at the forest edge, while Segment B transects were between 93 and 104 m long and spanned elevations between -0.255 and 1.117 m. The 11-year average percent of year inundated for each permanent plot varied with elevation, ranging from 0.64% to 65.93% at Segment A high and low marsh, respectively, and 0.76% to 70.74% at Segment B. Grouped across segments by marsh zone, permanent plots were inundated an average of 3.97% of the year in the high marsh and 42.92% in the low marsh, with the mid marsh plots (only found at Segment A) averaging 8.54% of the year underwater. There were significant linear increases in inundation time (calculated using hours inundated per year) for all plots at both Segments from 2009 to 2019 (S3 and S4 Figs). Inundation time increased at rates ranging from 8.83 to 129.33 hours per year at Segment A and 7.25 to 124.26 hours per year at Segment B over the 11-year sampling period. Rates of change generally increased with increasing proximity to the creek, and the highest rates of increase occurred in low marsh plots.

**Fig 3:**
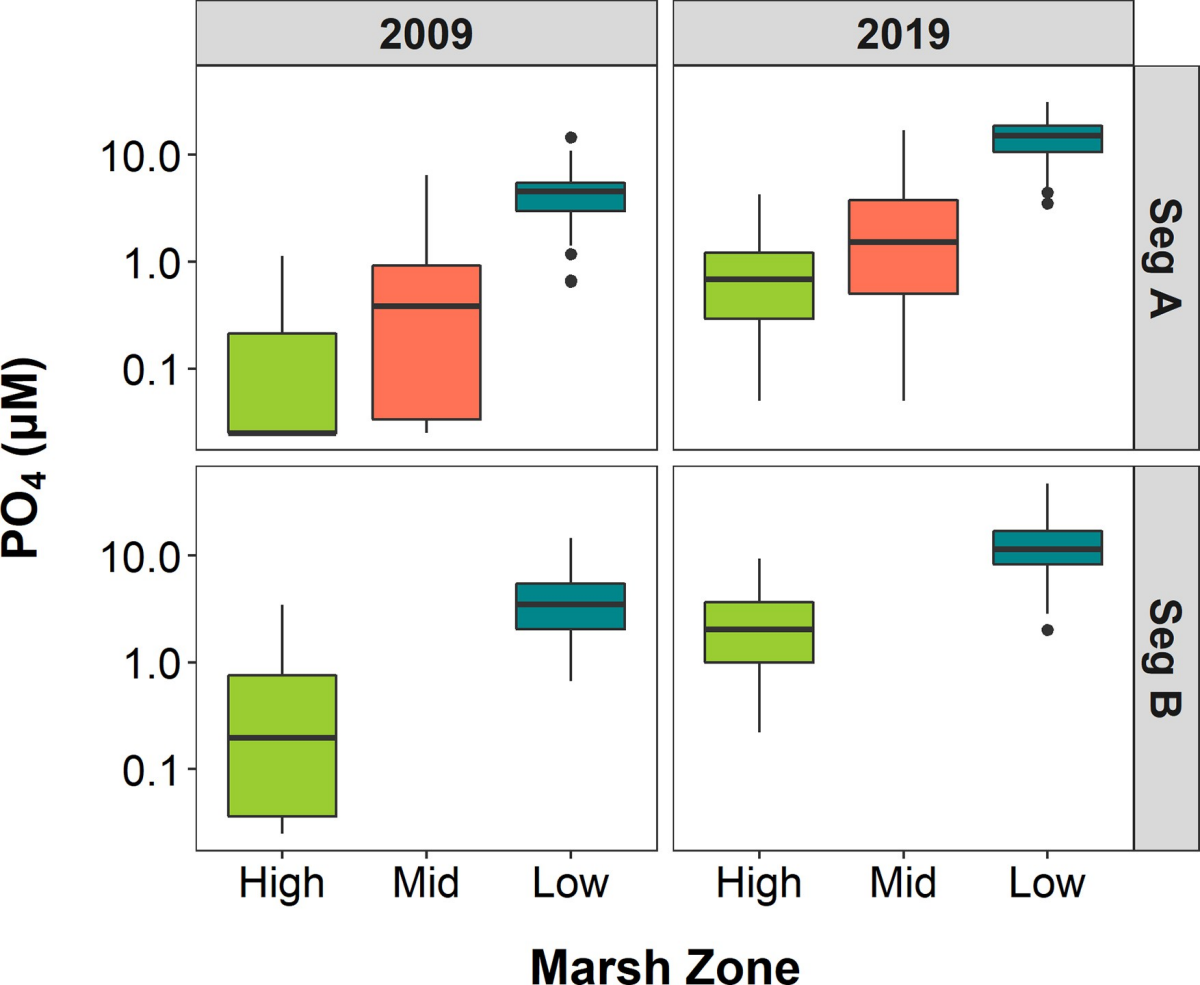
PO_4_ concentrations by marsh zone. Box and whisker plots of porewater PO_4_ concentrations across marsh zones in Segments A and B for years 2009 and 2019, with outliers included as points in line with whisker. The middle line of each box represents the median concentration, and the lower and upper lines the 25^th^ and 75^th^ percentiles. Whiskers extend to the most extreme data point no more than 1.5 the interquartile range. Permanent plots were grouped into high, mid or low marsh zones according to elevation and dominant vegetation type.

Mean PO_4_ concentrations differed between marsh zones and years at both segments, increasing from the high to the low marsh within a given year and increasing between 2009 and 2019 within each zone (Fig 3, Table 1). Mean PO_4_ concentrations within marsh zones were similar across both Segments for each year (Fig 3). There was a significant interaction between marsh zone and year at Segment B only (Table 1). The difference between high and low marsh zone mean PO_4_ concentrations across both Segments was 3.83 μM in 2009 and increased to 12.41 μM in 2019. Increases in mean PO_4_ concentration between 2009 and 2019 were highest in the low marsh zone, rising by 11.04 μM at Segment A and 9.36 μM at Segment B.

**Table 1:** Linear mixed effects model results.

Response	Segment	Factor	Num. DF	Den. DF	F	*p*	Marginal R^2^	Conditional R^2^
[PO_4_]	A	zone	2	184	166.90	<0.001	0.6647	0.6928
		year	1	6	25.44	0.002		
		zone:year	2	184	1.83	0.163		
	B	zone	1	198	328.06	<0.001	0.7392	0.6843
		year	1	6	30.97	0.001		
		zone:year	1	198	14.55	<0.001		
[NH_4_]	A	zone	2	184	28.10	<0.001	0.3543	0.308
		year	1	6	5.46	0.058		
		zone:year	2	184	9.25	<0.001		
	B	zone	1	198	272.12	<0.001	0.6745	0.652
		year	1	6	47.27	<0.001		
		zone:year	1	198	10.38	0.002		

Analysis of variance results from linear mixed effect model testing the effect of marsh zone (high, mid, low) and sampling year (2009 or 2019) on mean concentrations of PO_4_ and NH_4_ at the two marsh segments (A and B). Each model included marsh zone, year and the interaction between zone and year as fixed effects, while sampling month nested within year was included as a random effect. The marginal R^2^ indicates the variance explained by only the fixed effects, while the conditional R^2^ reflects the variance explained when the random effect is included in the model.

**Fig 4:**
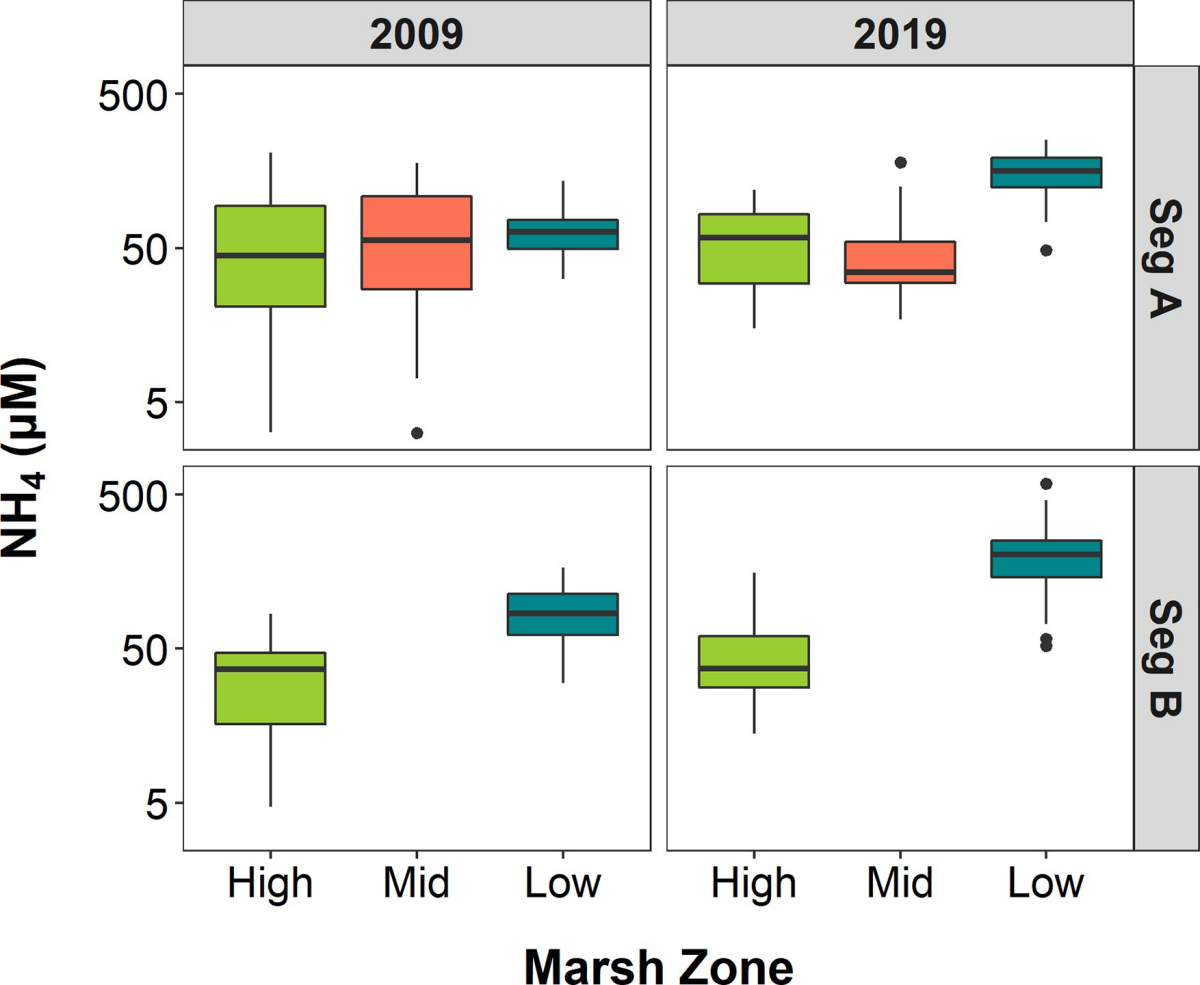
NH_4_ concentrations by marsh zone. Box and whisker plots of porewater NH_4_ concentrations across marsh zones in Segments A and B for years 2009 and 2019, with outliers included as points in line with whisker. The middle line of each box represents the median concentration, and the lower and upper lines the 25^th^ and 75^th^ percentiles. Whiskers extend to the most extreme data point no more than 1.5 the interquartile range. Permanent plots were grouped into high, mid or low marsh zones according to elevation and dominant vegetation type.

Spatial and temporal patterns in porewater NH_4_ concentrations were similar to the trends in PO_4_, with the highest quantities and largest increases generally occurring in the low marsh zone (Fig 4). The interaction between marsh zone and year was significant at both segments as were differences in the means across marsh zones (Table 1). The fixed effect of year on NH_4_ concentrations was only significant at Segment B. Mean NH_4_ concentrations in the low marsh zone across both Segments increased from 54.90 μM in 2009 to 191.50 μM in 2019. In 2009, concentrations in low marsh plots ranged from 29.75 μM to 167.38 μM while the range in 2019 was 48.45 μM to 589.99 μM.

**Fig 5:**
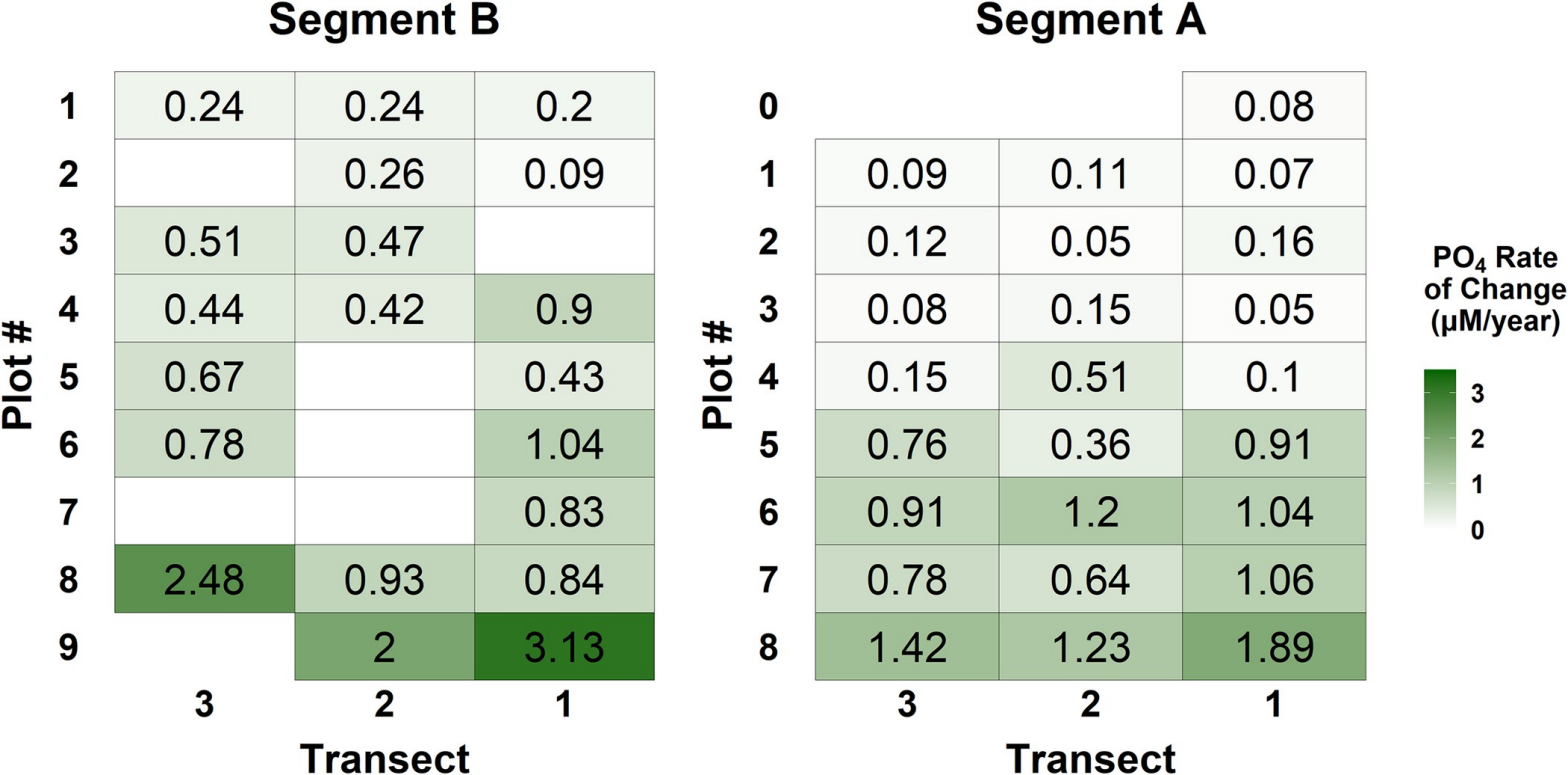
PO_4_ rates of change. Rates of change in PO_4_ (μmol L^-1^) corresponding to each permanent vegetation monitoring plot across Segments A and B. The numbers in each plot represent the slope of the linear fit between average monthly porewater PO_4_ concentration (from May-August) and years from 2009–2019 where *p* < 0.05. Plot shading visualizes the spatial variability in rates of change, and plots with no number/shading had non-significant temporal trends. Segment B is upstream and west of Segment A, so they are plotted here as they are oriented in the field.

Porewater PO_4_ concentrations increased across much of the marsh platform at both Segments from 2009 to 2019. Significant linear increases in porewater PO_4_ concentrations were found at 45 of the 51 permanent plots, with estimated rates of increase ranging from 0.05 to 3.13 μM/year (Fig 5, S5 and S6 Figs). The rates of change tended to increase with plot proximity to the creek, with the highest rates occurring in the plots immediately adjacent to the creek along each of the six transects. However, spatial variability occurred within this general trend as some plots further from the creek bank depicted higher rates of change than plots closer to the creek in the same transect, and three consecutive low marsh plots along the same transect at Segment B did not exhibit significant increases in PO_4_ concentrations over time (Fig 5).

**Fig 6:**
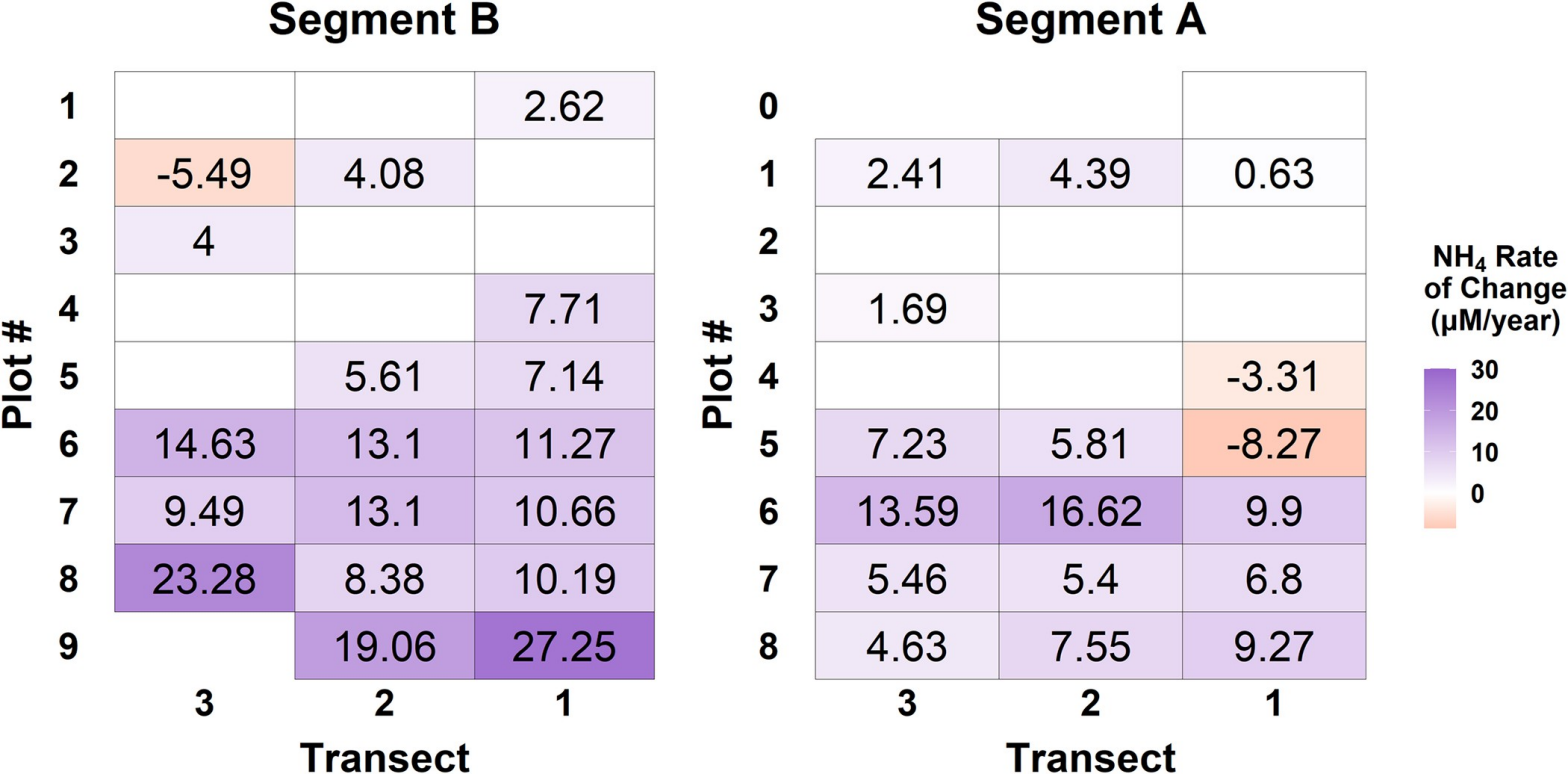
NH_4_ rates of change. Rates of change in NH_4_ (μmol L^-1^) corresponding to each permanent vegetation monitoring plot across Segments A and B. The numbers in each plot represent the slope of the linear fit between average monthly porewater NH_4_ concentration (from May-August) and years from 2009–2019 where *p* < 0.05. Plot shading visualizes the spatial variability in rates of change, and plots with no number/shading had non-significant temporal trends. Segment B is upstream and west of Segment A, so they are plotted here as they are oriented in the field.

Compared to the clear increases observed in porewater PO_4_, trends in porewater NH_4_ concentrations were more variable. Linear fits of NH_4_ concentrations as a function of year were statistically significant at 35 of 51 permanent plots with positive relationships at 32 plots and negative relationships at three plots (Fig 6, S7 and S8 Figs). Rates of change ranged from -8.27 to 16.62 μM/year at Segment A and -5.49 to 27.25 μM/year at Segment B. The highest rates of increase in NH_4_ concentrations occurred in low marsh plots. However, there was substantial spatial variability across the marsh platform, and the relationship between increasing distance from the forest and rate of change was inconsistent, with creek-side plots exhibiting the highest increases in NH_4_ on some transects and plots in the mid-marsh showing the largest change on other transects.

Porewater salinities declined across most of the marsh platform at both Segments from 2009 to 2019 (S9–S11 Figs). Significant linear decreases in porewater salinity were found at 40 of the 51 permanent plots, with estimated rates of decrease ranging from -0.47 to -1.99 psu/year across both Segments. There were no significant linear increases in porewater salinity at any of the permanent plots. There was no clear pattern in the magnitude of rates of decrease in salinity in terms of position along the marsh elevation gradient as observed with the rates of increase in nutrient concentrations. Particularly at Segment B, the rates of decrease in salinity are highly similar throughout the marsh. The plots that did not exhibit significant decreases over time in salinity were largely restricted to Segment A high and mid marsh plots located above Mean Higher High Water (MHHW).

**Fig 7:**
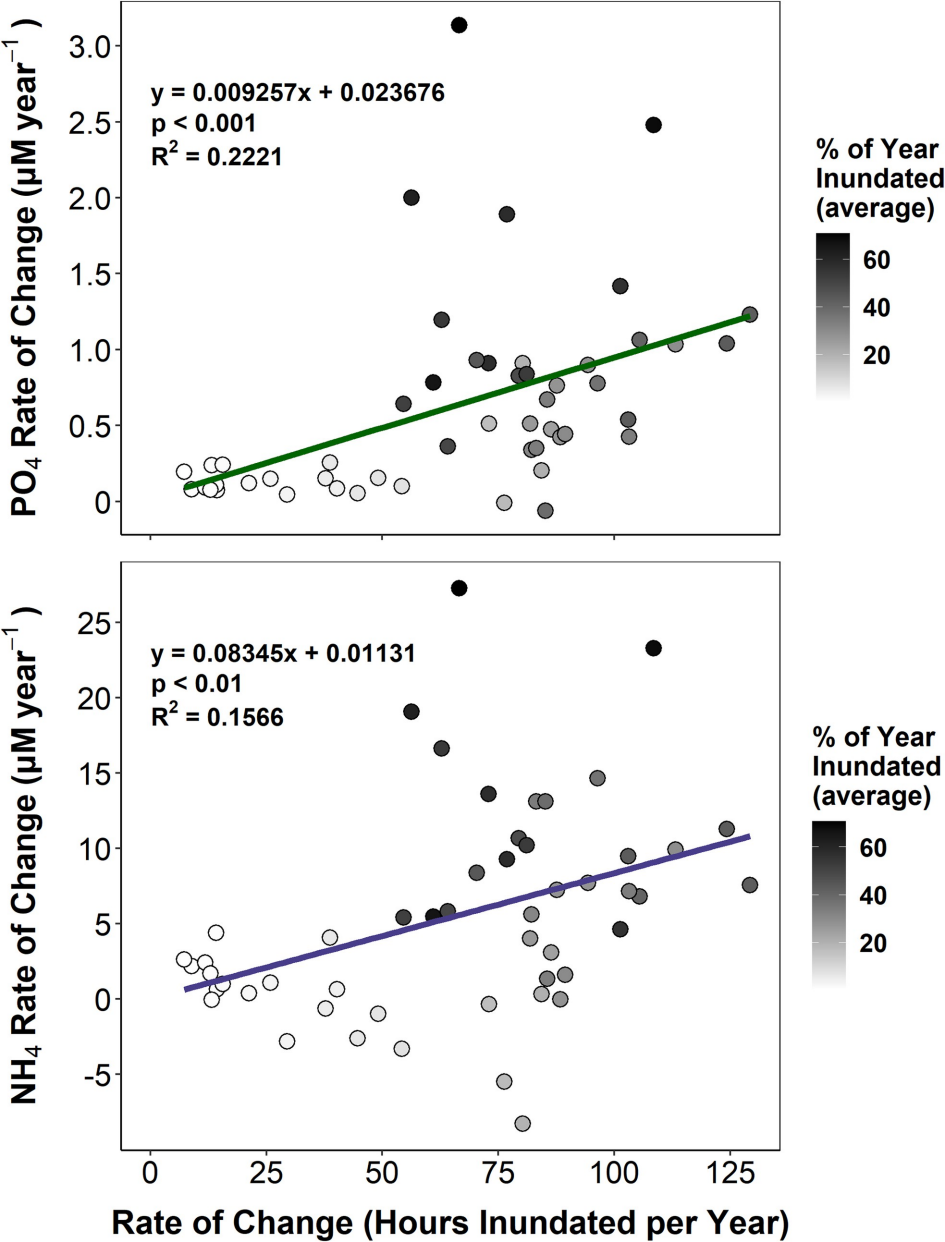
Rates of change in nutrients and inundation time. Linear fits between rates of change in inundation time (in hours inundated per year) and rates of change in porewater PO_4_ (A) and NH_4_ (B) concentrations across all permanent monitoring plots in Segments A and B. Each point corresponds to a permanent plot and is shaded according to the average of the percentages of each year the plots were inundated over the entire 2009–2019 period. These analyses include the rates of change calculated from all plot-level linear regressions between nutrient concentrations and year.

Rates of change in both NH_4_ and PO_4_ concentrations increased with the rate of change in inundation time, though changing inundation time explained slightly more variation in rates of change for PO_4_ (p < 0.001, R^2^ = 0.2221) than for NH_4_ (p < 0.01, R^2^ = 0.1566) (Fig 7). In the case of both NH_4_ and PO_4_, a rate of change in inundation time greater than approximately 50 hours per year was associated with greater variability in the rates of change in nutrient concentrations.

## Discussion

Changes to porewater nutrient concentrations in this representative southeastern U.S. salt marsh demonstrate clear spatial and temporal trends that correspond to increases in inundation time induced by sea-level rise. Over the course of the time series, NH_4_ and PO_4_ concentrations were consistently highest in the low marsh, where long-term average inundation times are commonly greater than ~40% of the year. In many instances, nutrient concentrations differed by an order of magnitude between high and low marsh plots. In addition to the consistent overall gradient of increasing nutrient concentrations with increasing proximity to the tidal creek over the course of sampling period, NH_4_ and PO_4_ concentrations generally increased across the entire marsh platform. Rates of increase of both nutrient parameters were generally highest in the low marsh at plots where inundation time has increased the most over the course of the time series. Significant but smaller increases in porewater NH_4_ and PO_4_ have occurred in many high and mid marsh plots where rates of increases in inundation time are reduced compared with the low marsh. Within both the spatial pattern of nutrient availability across marsh zones and the temporal trend of increasing concentrations through time, we observed small-scale spatial variability that we attribute to the complexity of interacting physical, biological and edaphic variables in salt marsh processes [23].

Concentrations measured in porewater equilibrators are the net result of local production, consumption, and transport processes integrated over the course of the deployment. As such, it is difficult to infer mechanisms controlling spatial patterns. Studies focusing on the spatial patterns of nutrient concentrations within a salt marsh system are commonly discussed in the context of factors relating to vegetation growth and distribution. Most work investigating trends in nutrient availability has focused on NH_4_ because growth of vegetation within southeastern US salt marshes is typically nitrogen-limited, and NH_4_ is the dominant form of inorganic nitrogen available in marsh sediments [19, 44, 45]. Lower NH_4_ concentrations are often observed adjacent to creek banks, where tall-form *S*. *alterniflora* is dominant, relative to interior areas of the marsh where short-form *S*. *alterniflora* is abundant [24, 25, 46, 47]. This pattern has primarily been attributed to the variable degree of soil flushing and drainage in the two areas, which facilitates more oxidized or reduced soil conditions [48]. More frequent tidal flooding of the marsh in close proximity to the creek bank promotes regular flushing of the porewaters and more oxidized soil conditions immediately adjacent to the creek bank. In turn, this enables vegetation in this area to readily take up available nutrients, such that little accumulation occurs [19]. More oxidized conditions also strongly promote PO_4_ adsorption by iron-oxides, resulting in lower porewater concentrations [47, 49]. Diminished soil drainage inland of the creek bank berm results in longer porewater residence times and fosters reduced conditions, hindering NH_4_ uptake efficiency in vegetation growth and iron-phosphate mineral formation. Based on our groupings of permanent plots into marsh zones, we found that nutrient concentrations were considerably higher in the low marsh zone than in the high and mid marsh zones. Importantly, our permanent plot classification regime did not distinguish between short and tall form *S*. *alterniflora* zones, both of which were included in the low marsh grouping based on their location along the elevation gradient. Thus, high concentrations in the more inland plots containing short-form *S*. *alterniflora* could have inflated the zone-wide average compared to if the low marsh zone was exclusively made up of tall-form *S*. *alterniflora*. Additionally, and in contrast to these previous studies, our high marsh plots represent a diversity of plant species composition. Observations from a salt marsh in Sapelo Island, GA, USA indicate that NH_4_ concentrations are lower in areas of the marsh interior primarily colonized by *Juncus roemarianus* compared with those dominated by short-form *S*. *alterniflora* at a similar elevation [25]. This suggests that vegetation type influences nutrient availability and could partially explain our findings that nutrient concentrations were lower in the high and mid marsh zones, where many plots are dominated by *J*. *roemarianus*, relative to the low marsh zone exclusively colonized by *S*. *alterniflora*. Finally, our low marsh, *S*. *alterniflora* dominated, zone tends to have higher organic matter concentrations and lower porosity, compared to the mid- and high-marsh [50]. Combined with the saturated soils and reducing conditions of this interior low marsh, this likely fuels higher rates of anaerobic metabolism and associated nutrient regeneration [51], which could also promote high concentrations in this region. This is consistent with previous studies showing higher porewater nutrient concentrations in lower, more frequently flooded areas of the marsh platform [52].

Small-scale differences in sediment characteristics, flooding frequency and duration, and plant distributions may contribute to some of the variability we observed in the temporal trends in nutrient availability at the plot scale. Across the marsh platform, we observed a general positive relationship between rate of change in plot inundation time (in hours per year inundated) and the rate of increase in porewater nutrient concentrations. However, in select plots primarily located in the mid and high marsh zones, there was no statistically significant trend for one or both nutrient parameters, and decreases in NH_4_ over time were observed in three plots. Although conventional models of salt marsh zonation depict relatively monotonic changes in inundation and plant zonation with increasing elevation, patchy microtopography and small-scale variability in vegetation distributions and growth are a common in many marshes [53]. Patchiness in inundation, vegetation, and sediment physical characteristics (e.g. porosity, grain size) across the mid and high marsh likely result in variable soil drainage and flushing, driving changes in nutrient biogeochemistry [54]. This likely promotes small-scale variability in porewater nutrient concentrations by many of the same conditions and mechanisms discussed above for variability along the marsh platform gradient. An additional potential explanation for the variability observed in our temporal trends is increased flushing in areas of the high and mid marsh zones due to rising water levels. This may promote newly oxidized conditions under which plants can better utilize available nutrients for growth, thereby resulting in reduced nutrient accumulation in sediment porewater. At the same time, increased flushing, and therefore more oxidized conditions, could promote plant growth and in turn increase organic matter supply to the sediments, which could result in increased NH_4_ accumulation as it decomposes. Finally, nutrient uptake by salt marsh vegetation varies by species and is influenced by interspecific competition and salinity, so variability in resident plant communities as well as porewater salinity may explain differences in concentrations of NH_4_ and PO_4_ [22, 55, 56]. In spite of the potential variability resulting from the interplay between sedimentary conditions, hydrology, and vegetation growth, we observed a clear overall trend of increasing nutrient concentrations across the marsh platform over time. We hypothesize that more frequent and prolonged inundation enhances sediment flushing with surface water, which intensifies redox conditions and organic matter cycling processes that foster and maintain higher nutrient concentrations via complex feedbacks between hydrology and biogeochemistry.

Increasing NH_4_ and PO_4_ concentrations in salt marsh porewater may be directly relevant to water quality throughout the broader estuarine system given that porewater flux is a key mode of nutrient transport from salt marshes into tidal creeks [57–59]. Hydraulic gradients driven by tidal inundation and draining drive a process known as tidal pumping, in which marsh porewaters drain from the salt marsh platform into adjacent creeks at low tide [26]. Furthermore, modeling demonstrates that increased tidal inundation at higher elevations enhances this tidal pumping mechanism, resulting in increased volumes of porewater flux to adjacent marsh creeks [27]. Concentrations of NH_4_ and PO_4_ are higher in salt marsh porewaters compared with adjacent tidal creeks, often by an order of magnitude, so small flux volumes moving from the marsh into creeks can have outsized effects on water column nutrient budgets [27, 28]. Given that porewater nutrient concentrations have increased by nearly an order of magnitude in many areas of the low marsh over the course of our study, the importance of porewater flux to water column nutrient budgets is also likely increasing. Additionally, as more of the marsh platform becomes frequently inundated at high tide due to sea level rise, compression of marsh sediments and overall increase of porewater exchange due increased sediment-surface water contact will further enhance nutrient flux from the marsh sediments into tidal creeks [59]. Thus, sea level rise may be implicated in the eutrophication of the estuarine water column, even within otherwise unimpacted ecosystems such as the North Inlet estuary, though this remains to be investigated.

Similarly, higher concentrations of NH_4_ and PO_4_ in marsh porewater may have implications for marsh stability via effects on vegetation growth, which may in turn influence the vulnerability of marshes to sea level rise. Studies examining the response of marsh vegetation to increased nutrient availability via addition of fertilizers (e.g., dissolved inorganic nitrogen and phosphorus), report conflicting results on plant growth. Admittedly, nutrient addition via fertilization may have different effects on vegetation dynamics than increased nutrient availability due to biogeochemical changes associated with inundation. Nevertheless, increased nutrient availability via fertilization can result in decreased belowground biomass of *S*. *alterniflora* due to the reduced need for extensive root and rhizome networks to acquire required quantities of nutrients [22, 60–62]. In turn, reduced belowground biomass, which binds and stabilizes sediments, may lead to increased vulnerability of creek banks to erosion and collapse. Within a salt marsh in a neighboring subbasin of North Inlet estuary, Wigand and colleagues [34] demonstrated a decrease in fine root biomass in response to fertilization, although this was coincident with an increase in belowground coarse roots, rhizomes and organic matter. They attribute the latter result to the minerogenic nature of the system, suggesting that sedimentation processes in a given salt marsh influence how increased nutrient availability affects belowground biomass. In other cases, increased nutrient availability has resulted in increased aboveground biomass, which may enhance sediment capture and marsh accretion, thereby improving the ability of the marsh to keep pace with rising sea level [22, 61, 63]. Finally, in a nutrient enrichment study of the salt marsh at Sapelo Island, fertilization resulted in increased aboveground biomass of *S*. *alterniflora* and enhanced competition by *S*. *alterniflora* in typically mixed vegetation areas, suggesting that increased nutrient availability can drive shifts in marsh plant zonation [21]. Thus, inundation-driven increases in porewater nutrient concentrations may alter how plants allocate resources for growth and stimulate ecosystem restructuring, which is relevant to marsh stability in the face of sea level rise.

The observational nature of this study limits our ability to investigate the mechanisms underlying the observed spatiotemporal patterns in salt marsh porewater nutrient concentrations. Nevertheless, the absence of any development or major changes in land use over the course of our sampling period indicate that the increasing trend in nutrient concentrations is not a direct anthropogenic impact. An alternative hypothesis that could explain increasing nutrient concentrations within marsh porewaters is enhanced runoff or precipitation-driven shallow groundwater flow from the adjacent forested watershed, which would be consistent with our results indicating decreasing salinities across the marsh platform [64]. However, precipitation data collected at the NI-WB Oyster Landing Meteorological Station, which is at the mouth of Crabhaul Creek, do not exhibit a significant increasing trend over the 11- year sampling period (S12 Fig) [65]. As such, we do not believe enhanced input of nutrients from the forested uplands is a major factor driving the observed changes. We also considered that enhanced evaporation could drive increases in porewater nutrient concentrations; however, decreasing salinities across most of the marsh platform over the sampling period directly contradict this potential explanation (S9–S11 Figs) [42, 66]. Moreover, our observations of highest nutrient increases occurring in the low marsh are the opposite of what would be expected if evaporation was driving the changes, given that evaporation is greater in the marsh interior due to variability in sediment characteristics and reduced flooding relative to the low marsh [67, 68]. Our data are most consistent with the hypothesis that increasing inundation time, by acting on biogeochemical processes within marsh sediments, is the primary driver of internal cycling processes that result in the drastic increases in porewater NH_4_ and PO_4_ documented here. Furthermore, we would expect increasing inundation to be associated with decreasing salinities across the marsh platform, as higher-salinity porewaters (resulting from high evapotranspiration), particularly in the marsh interior, are increasingly diluted with relatively lower-salinity creekwater and increased flooding increasingly inhibits evaporation [42]. Remarkably, we were unable to find any published long-term datasets on porewater nutrient concentrations in other salt marsh systems for comparison with our findings and evaluation of the hypothesized link between sea level rise and rising nutrient concentrations across different locations. However, preliminary analyses of unpublished data available from the Plum Island marsh LTER site and Goal Island marsh in North Inlet are suggestive of increasing trends in porewater nutrient concentrations that are consistent with our observations at Crabhaul Creek marsh [69, 70].

The observed relationship between increased interstitial nutrient concentrations and changing inundation patterns across the salt marsh platform due to sea level rise presents several opportunities for future research. Data on other parameters relevant to salt marsh biogeochemistry (e.g., oxidation-reduction potential, porewater iron, sulfide content) may provide insights into the processes underlying the aforementioned relationship as well as the small-scale spatial variability detected within the broader patterns. Furthermore, the burrows of marsh crabs can act as conduits for oxygen in otherwise largely anoxic sediments and as interfaces of active solute exchange [71, 72]; however, the relationship between crab burrow density and spatial patterns in biogeochemistry remains largely unexplored. Additionally, investigating long-term trends in surface water nutrients in North Inlet estuary will provide insight into whether enhanced tidal pumping of marsh porewater, and therefore nutrient export, has led to increases in water column nutrient concentrations. Finally, direct quantification of nutrient flux via porewater exchange at the sediment-surface water interface (*i*.*e*. via seepage meters [28]) would enable us to determine baseline estimates of salt marsh nutrient inputs to the water column, which is critical for understanding factors regulating nutrient budgets in estuarine systems. This study demonstrates the value of rigorous monitoring programs for detecting and describing environmental changes associated with sea level rise as well as anticipating how coastal ecosystems will respond to changing climatic and hydrologic regimes.

## Supporting information

S1 Table Plot classifications for statistical analysis. Data on marsh zone classification, distance from forest, elevation, and dominant vegetation type at each permanent vegetation monitoring plot. Mixed vegetation plots include more than two species common to mid and high marsh elevations, such as *I*. *frutescens*, *B*.*frutescens*, *S*. *patens*, *D*. *spicatta*, *J*. *roemarianus*, *S*. *tenuiflorium*, and *I*. *sagittata*.
(DOCX)

S1 Fig Local sea-level trends. Monthly mean sea-level (relative to the Mean Sea Level datum established by NOAA CO-OPS) at Charleston, SC detrended for seasonal fluctuations. The blue line represents the long-term rate of sea-level rise (3.39 mm/year), while the red line depicts the rate of increase in mean sea-level from 2009–2019 (13.2 mm/year). The rate of sea-level change from 2009–2019 was calculated using a linear regression in which year was the predictor variable and monthly mean sea-level was the response (*p* < 0.001).
(TIF)

S2 Fig Study site and sampling design. Oblique aerial photographs of Crabhaul Creek at marsh Segment A (top) and Segment B (bottom) taken using a drone from 100m height on 5/2/21. Locations of permanent plots along the 6 transects (3 at Segment A, 3 at Segment B) are denoted by yellow arrows.
(TIF)

S3 Fig Plot inundation times at Segment A. Total number of hours inundated for every year of the sampling period (2009–2019) at all permanent plots in Segment A. The transects at each Segment (3, 2, 1) are indicated at the top of figure, while the individual plot numbers (0–8) are indicated at the right hand side. Permanent plots with significant linear relationships (*p* < 0.05) between inundation time (hours inundated) and year include a best fit line on the figure which depicts the slope of the regression.
(TIF)

S4 Fig Plot inundation times at Segment B. Total number of hours inundated for every year of the sampling period (2009–2019) at all permanent plots in Segment B. The transects at each Segment (3, 2, 1) are indicated at the top of figure, while the individual plot numbers (1–9) are indicated at the right hand side. Permanent plots with significant linear relationships (*p* < 0.05) between inundation time (hours inundated) and year include a best fit line on the figure which depicts the slope of the regression.
(TIF)

S5 Fig PO_4_ concentrations at Segment A. Monthly average PO_4_ concentrations (μM) for every year of the sampling period (2009–2019) at all permanent plots in Segment A. The transects at each Segment (3, 2, 1) are indicated at the top of figure, while the individual plot numbers (0–8) are indicated at the right hand side. Permanent plots with significant linear relationships (*p* < 0.05) between PO_4_ concentration and year include a best fit line on the figure which depicts the slope of the regression.
(TIF)

S6 Fig PO_4_ concentrations at Segment B. Monthly average PO_4_ concentrations (μM) for every year of the sampling period (2009–2019) at all permanent plots in Segment B. The transects at each Segment (3, 2, 1) are indicated at the top of figure, while the individual plot numbers (1–9) are indicated at the right hand side. Permanent plots with significant linear relationships (*p* < 0.05) between PO_4_ concentration and year include a best fit line on the figure which depicts the slope of the regression.
(TIF)

S7 Fig NH_4_ concentrations at Segment. A. Monthly average NH_4_ concentrations (μM) for every year of the sampling period (2009–2019) at all permanent plots in Segment A. The transects at each Segment (3, 2, 1) are indicated at the top of figure, while the individual plot numbers (0–8) are indicated at the right hand side. Permanent plots with significant linear relationships (*p* < 0.05) between NH_4_ concentration and year include a best fit line on the figure which depicts the slope of the regression.
(TIF)

S8 Fig NH_4_ concentrations at Segment B. Monthly average NH_4_ concentrations (μM) for every year of the sampling period (2009–2019) at all permanent plots in Segment B. The transects at each Segment (3, 2, 1) are indicated at the top of figure, while the individual plot numbers (1–9) are indicated at the right hand side. Permanent plots with significant linear relationships (*p* < 0.05) between NH_4_ concentration and year include a best fit line on the figure which depicts the slope of the regression.
(TIF)

S9 Fig Salinity rates of change. Rates of change in salinity (psu) corresponding to each permanent vegetation monitoring plot across Segments A and B. The numbers in each plot represent the slope of the linear fit between average monthly porewater salinities (from May-August) and years from 2009–2019 where *p* < 0.05. Plot shading visualizes the spatial variability in rates of change, and plots with no number/shading had non-significant temporal trends. Segment B is upstream and west of Segment A, so they are plotted here as they are oriented in the field.
(TIF)

S10 Fig Salinities at Segment A. Monthly average salinities (psu) for every year of the sampling period (2009–2019) at all permanent plots in Segment A. The transects at each Segment (3, 2, 1) are indicated at the top of figure, while the individual plot numbers (0–8) are indicated at the right hand side. Permanent plots with significant linear relationships (*p* < 0.05) between salinity and year include a best fit line on the figure which depicts the slope of the regression.
(TIF)

S11 Fig Salinities at Segment B. Monthly average salinities (psu) for every year of the sampling period (2009–2019) at all permanent plots in Segment B. The transects at each Segment (3, 2, 1) are indicated at the top of figure, while the individual plot numbers (1–9) are indicated at the right hand side. Permanent plots with significant linear relationships (*p* < 0.05) between salinity and year include a best fit line on the figure which depicts the slope of the regression.
(TIF)

S12 Fig Monthly precipitation anomaly recorded at Oyster Landing (North Inlet, SC), 2009–2019. The anomaly corresponds to deviation from the long-term mean monthly precipitation from 2009–2019 (gray line). A linear regression in which the predictor was date and the response was precipitation anomaly revealed no significant trend (F 1,130 = 1.792, *p* = 0.183). The highest spikes in the second half of the time series correspond to large tropical storm/hurricane events that took place after the summer vegetation growing season which is when porewater nutrients were measured.
(TIF)
