# Supplementary material for: Increasing tidal inundation corresponds to rising porewater nutrient concentrations in a southeastern U.S. salt marsh
Source: PLoS One. 2022 Nov 28;17(11):e0278215. doi: 10.1371/journal.pone.0278215 (PMC9704656; doi:10.1371/journal.pone.0278215)
Supplement: S1 Table — Data on marsh zone classification, distance from forest, elevation, and dominant vegetation type at each permanent vegetation monitoring plot. Mixed vegetation plots include more than two species common to mid and high marsh elevations, such as I. frutescens, B.frutescens, S. patens, D. spicatta, J. roemarianus, S. tenuiflorium, and I. sagittata. (DOCX) [file pone.0278215.s001.docx]

| **Segment** | **Transect** | **Plot #** | **Plot ID** | **Marsh Zone** | **Distance from Forest (m)** | **Elevation**  **(m NAVD 88)** | **Dominant Vegetation Species** |
| --- | --- | --- | --- | --- | --- | --- | --- |
| A | 1 | 0 | a10 | High | 0.00 | 1.09 | *I. frutescens, J. roemarianus* |
| A | 1 | 1 | a11 | High | 20.70 | 1.01 | *J. roemerianus* |
| A | 1 | 2 | a12 | High | 58.59 | 0.7747 | *J. roemerianus* |
| A | 1 | 3 | a13 | Mid | 93.28 | 0.782 | *S. depressa* |
| A | 1 | 4 | a14 | Mid | 130.66 | 0.745 | Mixed |
| A | 1 | 5 | a15 | Mid | 165.81 | 0.5463 | *S. alternifora* |
| A | 1 | 6 | a16 | Low | 201.29 | 0.4073 | *S. alternifora* |
| A | 1 | 7 | a17 | Low | 236.67 | 0.2423 | *S. alternifora* |
| A | 1 | 8 | a18 | Low | 273.36 | -0.0397 | *S. alternifora* |
| A | 2 | 1 | a21 | High | 4.03 | 1.0023 | *J. roemerianus* |
| A | 2 | 2 | a22 | High | 41.64 | 0.8877 | *S. depressa, L. carolinianum* |
| A | 2 | 3 | a23 | Mid | 78.21 | 0.8203 | Bare (Salt panne) |
| A | 2 | 4 | a24 | Mid | 115.84 | 0.578 | *S. depressa, S. alterniflora* |
| A | 2 | 5 | a25 | Low | 153.52 | 0.1303 | *S. alterniflora* |
| A | 2 | 6 | a26 | Low | 194.54 | 0.0673 | *S. alterniflora* |
| A | 2 | 7 | a27 | Low | 230.72 | 0.0877 | *S. alterniflora* |
| A | 2 | 8 | a28 | Low | 266.80 | 0.211 | *S. alterniflora* |
| A | 3 | 1 | a31 | High | 0.00 | 1.072 | Mixed |
| A | 3 | 2 | a32 | High | 25.07 | 0.9337 | Mixed |
| A | 3 | 3 | a33 | Mid | 61.24 | 1.019 | Mixed |
| A | 3 | 4 | a34 | Mid | 97.14 | 0.9037 | Mixed |
| A | 3 | 5 | a35 | Low | 132.66 | 0.4227 | *S. alterniflora* |
| A | 3 | 6 | a36 | Low | 174.57 | -0.005 | *S. alterniflora* |
| A | 3 | 7 | a37 | Low | 208.65 | -0.162 | *S. alterniflora* |
| A | 3 | 8 | a38 | Low | 244.66 | 0.0287 | *S. alterniflora* |
| B | 1 | 1 | b11 | High | 2.60 | 1.0717 | *J. roemerianus, C. jamaicense* |
| B | 1 | 2 | b12 | High | 11.33 | 0.8623 | *J. roemerianus* |
| B | 1 | 3 | b13 | High | 21.26 | 0.5363 | *S. alternifora* |
| B | 1 | 4 | b14 | Low | 27.04 | 0.4337 | *S. alternifora* |
| B | 1 | 5 | b15 | Low | 40.29 | 0.3613 | *S. alternifora* |
| B | 1 | 6 | b16 | Low | 53.31 | 0.247 | *S. alternifora* |
| B | 1 | 7 | b17 | Low | 67.73 | 0.1457 | *S. alternifora* |
| B | 1 | 8 | b18 | Low | 80.69 | 0.061 | *S. alternifora* |
| B | 1 | 9 | b19 | Low | 92.91 | -0.2533 | Bare (Mudflat) |
| B | 2 | 1 | b21 | High | 4.35 | 0.996 | Mixed |
| B | 2 | 2 | b22 | High | 12.23 | 0.783 | *S. alternifora, J. roemerianus* |
| B | 2 | 3 | b23 | Low | 26.54 | 0.4727 | *S. alternifora* |
| B | 2 | 4 | b24 | Low | 40.08 | 0.4163 | *S. alternifora* |
| B | 2 | 5 | b25 | Low | 52.54 | 0.3663 | *S. alternifora* |
| **Segment** | **Transect** | **Plot #** | **Plot ID** | **Marsh Zone** | **Distance from Forest (m)** | **Elevation**  **(m NAVD 88)** | **Dominant Vegetation Species** |
| B | 2 | 6 | b26 | Low | 64.95 | 0.3333 | *S. alternifora* |
| B | 2 | 7 | b27 | Low | 77.91 | 0.2803 | *S. alternifora* |
| B | 2 | 8 | b28 | Low | 90.10 | 0.2033 | *S. alternifora* |
| B | 2 | 9 | b29 | Low | 102.55 | -0.0377 | *S. alternifora* |
| B | 3 | 1 | b31 | High | 1.94 | 0.9793 | *J. roemerianus* |
| B | 3 | 2 | b32 | Low | 12.96 | 0.567 | *S. alternifora* |
| B | 3 | 3 | b33 | Low | 25.91 | 0.4387 | *S. alternifora* |
| B | 3 | 4 | b34 | Low | 40.89 | 0.3847 | *S. alternifora* |
| B | 3 | 5 | b35 | Low | 54.10 | 0.349 | *S. alternifora* |
| B | 3 | 6 | b36 | Low | 67.41 | 0.306 | *S. alternifora* |
| B | 3 | 7 | b37 | Low | 81.88 | 0.2157 | *S. alternifora* |
| B | 3 | 8 | b38 | Low | 93.66 | -0.208 | Bare (Mudflat) |

**S3 Table.**
