# Supplementary figures and images for: Increasing tidal inundation corresponds to rising porewater nutrient concentrations in a southeastern U.S. salt marsh
Source: PLoS One. 2022 Nov 28;17(11):e0278215. doi: 10.1371/journal.pone.0278215 (PMC9704656; doi:10.1371/journal.pone.0278215)

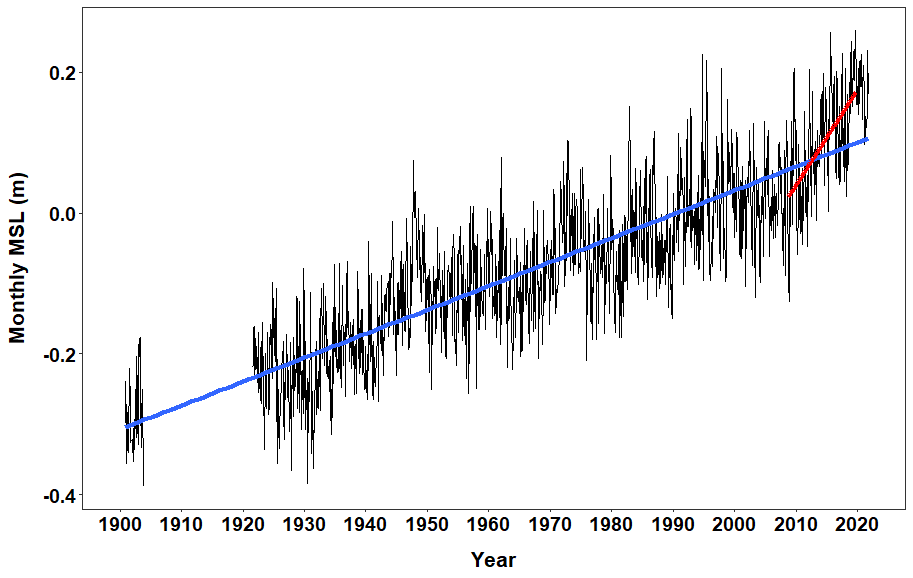

Supplement: S1 Fig — Monthly mean sea-level (relative to the Mean Sea Level datum established by NOAA CO-OPS) at Charleston, SC detrended for seasonal fluctuations. The blue line represents the long-term rate of sea-level rise (3.39 mm/year), while the red line depicts the rate of increase in mean sea-level from 2009–2019 (13.2 mm/year). The rate of sea-level change from 2009–2019 was calculated using a linear regression in which year was the predictor variable and monthly mean sea-level was the response (p < 0.001). (TIF) [file pone.0278215.s002.tif]

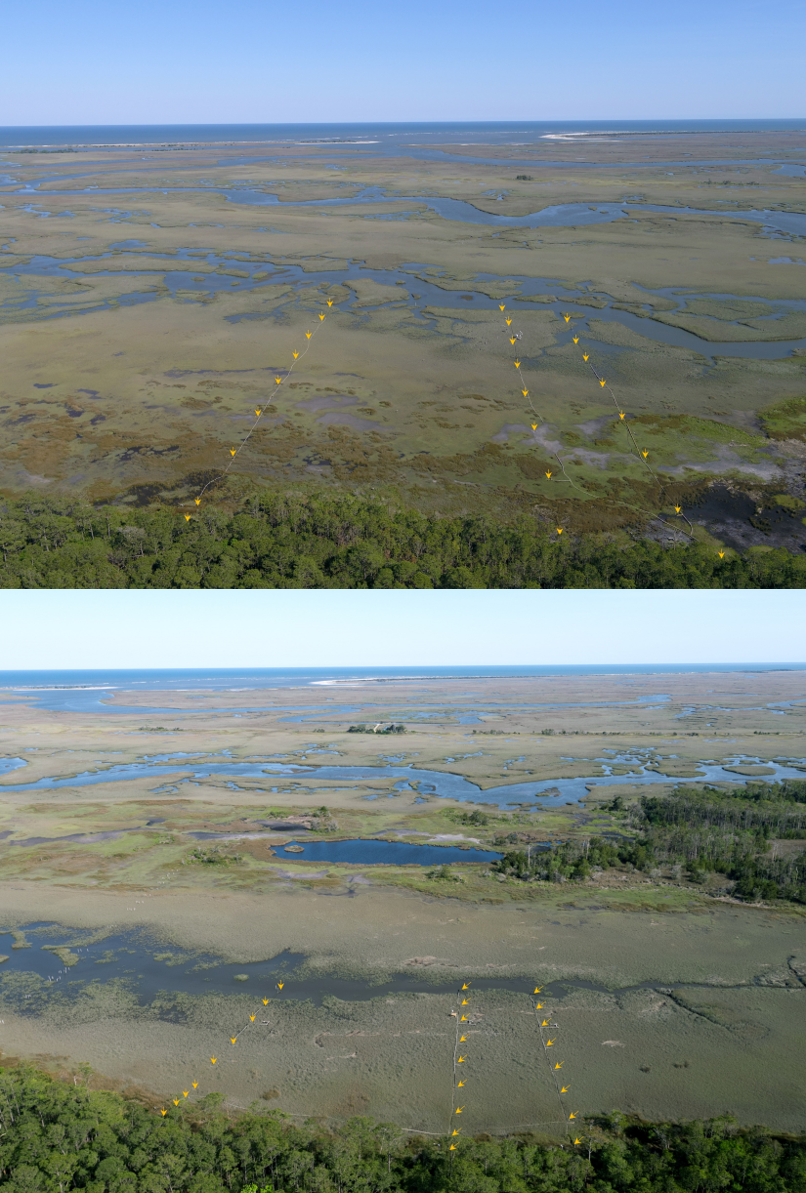

Supplement: S2 Fig — Oblique aerial photographs of Crabhaul Creek at marsh Segment A (top) and Segment B (bottom) taken using a drone from 100m height on 5/2/21. Locations of permanent plots along the 6 transects (3 at Segment A, 3 at Segment B) are denoted by yellow arrows. (TIF) [file pone.0278215.s003.tif]

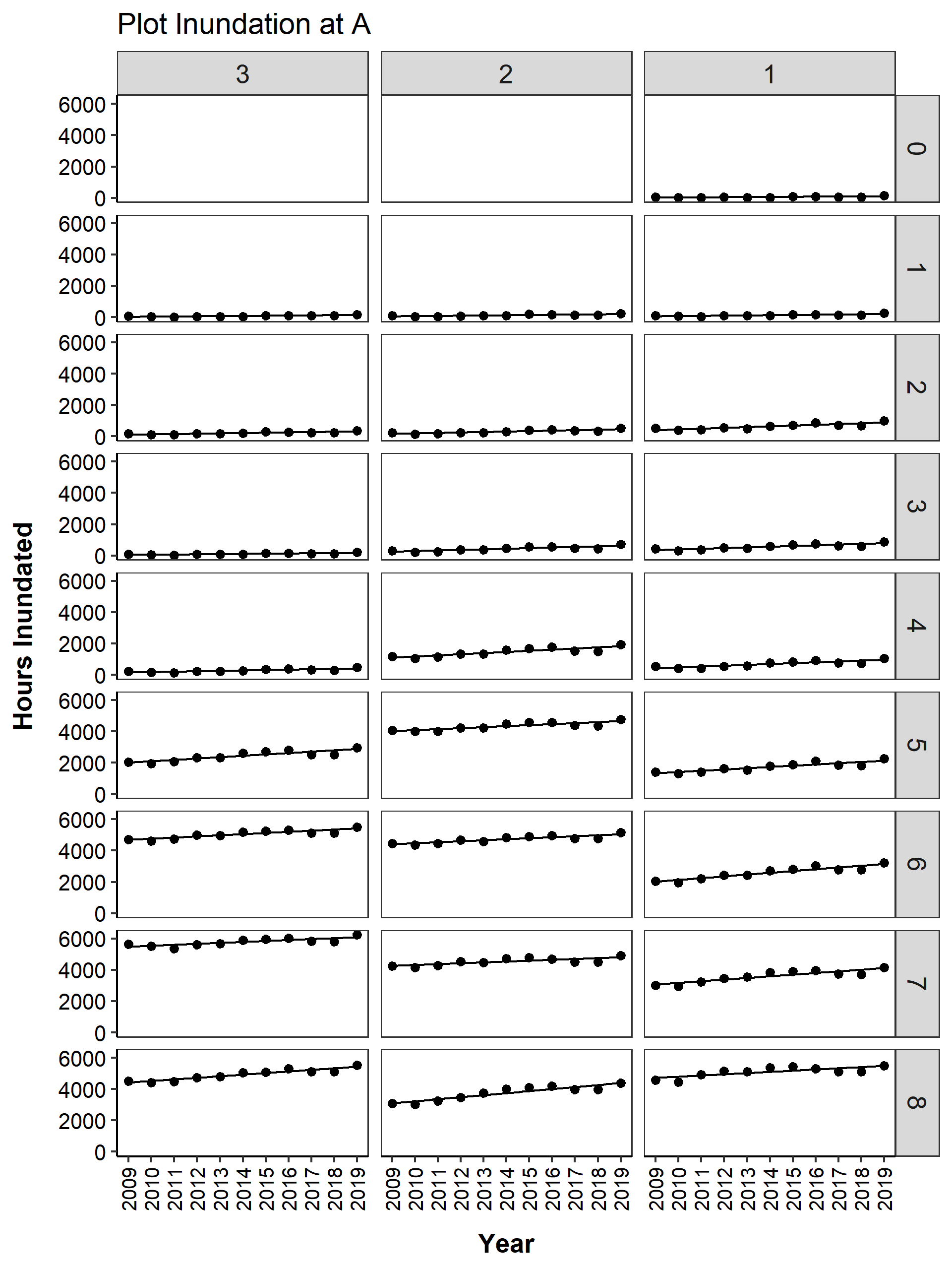

Supplement: S3 Fig — Total number of hours inundated for every year of the sampling period (2009–2019) at all permanent plots in Segment A. The transects at each Segment (3, 2, 1) are indicated at the top of figure, while the individual plot numbers (0–8) are indicated at the right hand side. Permanent plots with significant linear relationships (p < 0.05) between inundation time (hours inundated) and year include a best fit line on the figure which depicts the slope of the regression. (TIF) [file pone.0278215.s004.tif]

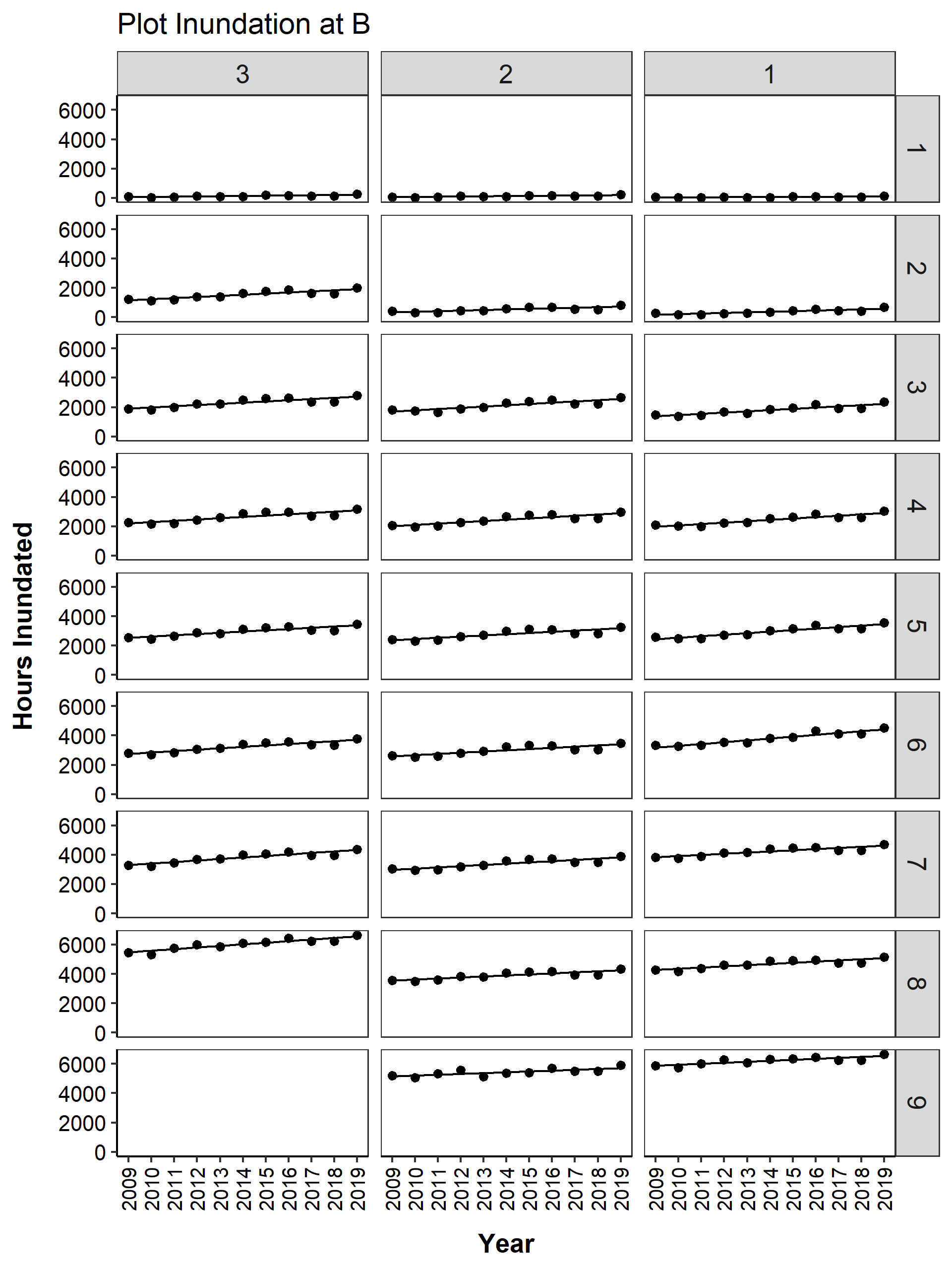

Supplement: S4 Fig — Total number of hours inundated for every year of the sampling period (2009–2019) at all permanent plots in Segment B. The transects at each Segment (3, 2, 1) are indicated at the top of figure, while the individual plot numbers (1–9) are indicated at the right hand side. Permanent plots with significant linear relationships (p < 0.05) between inundation time (hours inundated) and year include a best fit line on the figure which depicts the slope of the regression. (TIF) [file pone.0278215.s005.tif]

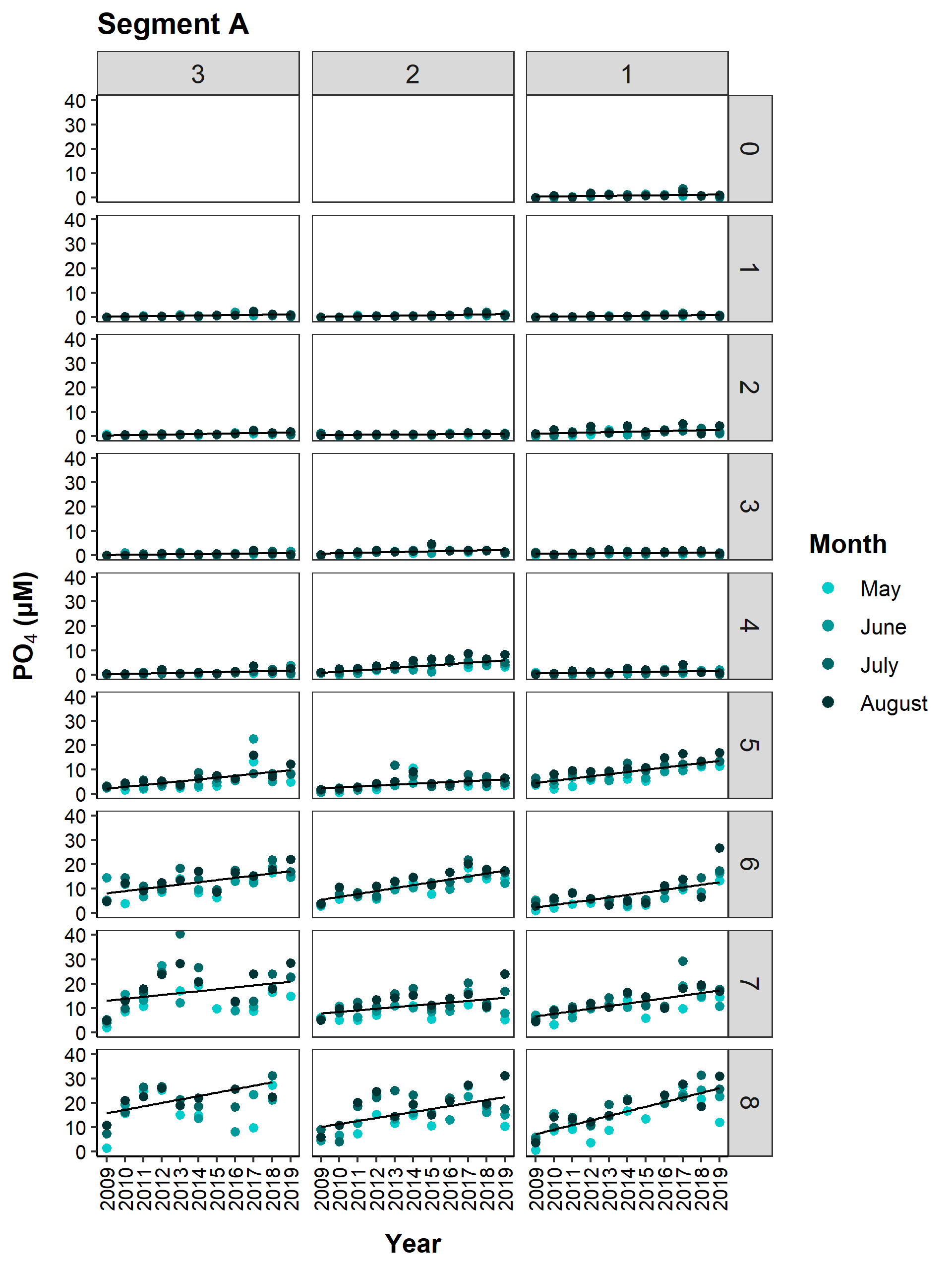

Supplement: S5 Fig — Monthly average PO4 concentrations (μM) for every year of the sampling period (2009–2019) at all permanent plots in Segment A. The transects at each Segment (3, 2, 1) are indicated at the top of figure, while the individual plot numbers (0–8) are indicated at the right hand side. Permanent plots with significant linear relationships (p < 0.05) between PO4 concentration and year include a best fit line on the figure which depicts the slope of the regression. (TIF) [file pone.0278215.s006.tif]

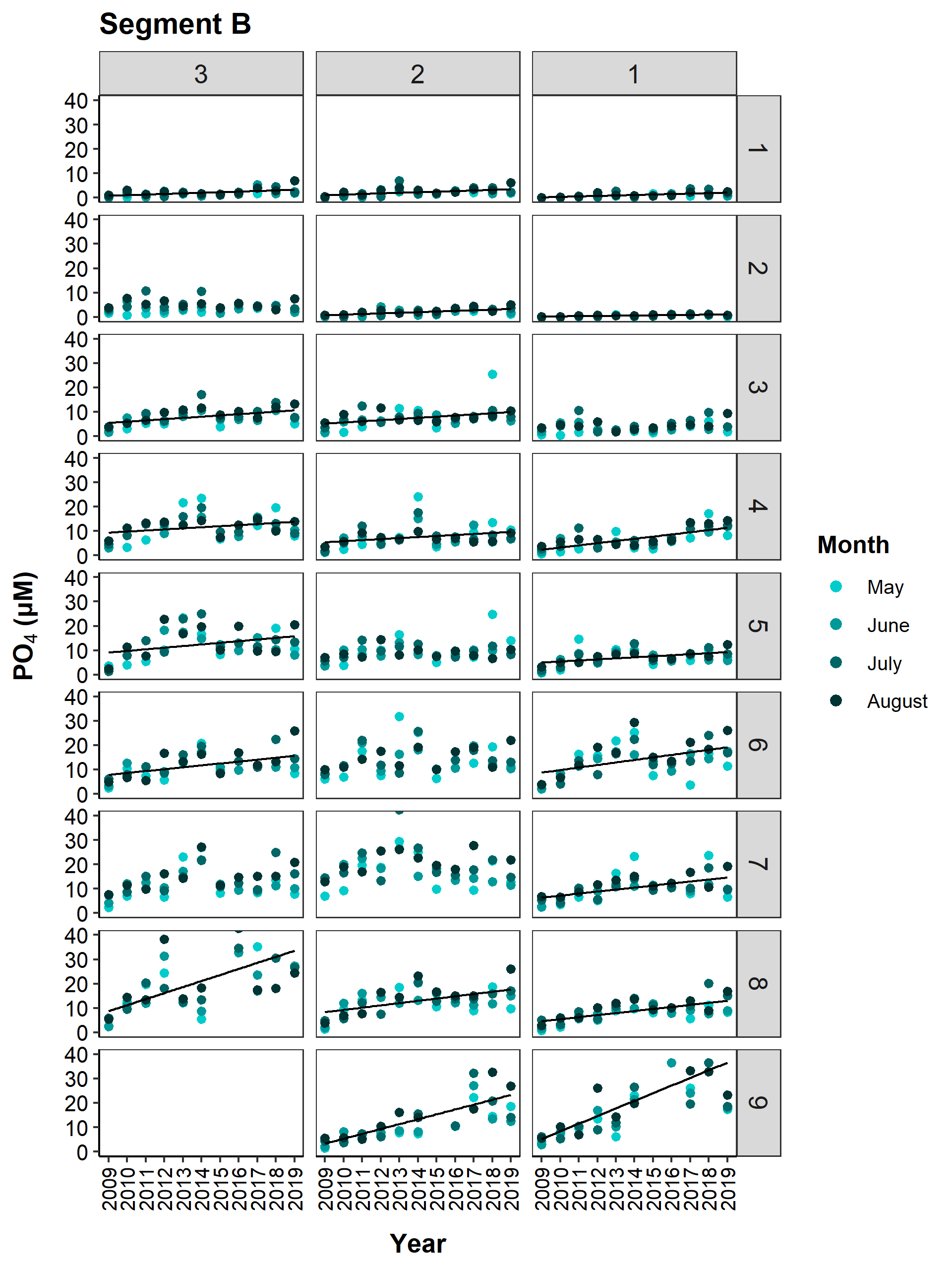

Supplement: S6 Fig — Monthly average PO4 concentrations (μM) for every year of the sampling period (2009–2019) at all permanent plots in Segment B. The transects at each Segment (3, 2, 1) are indicated at the top of figure, while the individual plot numbers (1–9) are indicated at the right hand side. Permanent plots with significant linear relationships (p < 0.05) between PO4 concentration and year include a best fit line on the figure which depicts the slope of the regression. (TIF) [file pone.0278215.s007.tif]

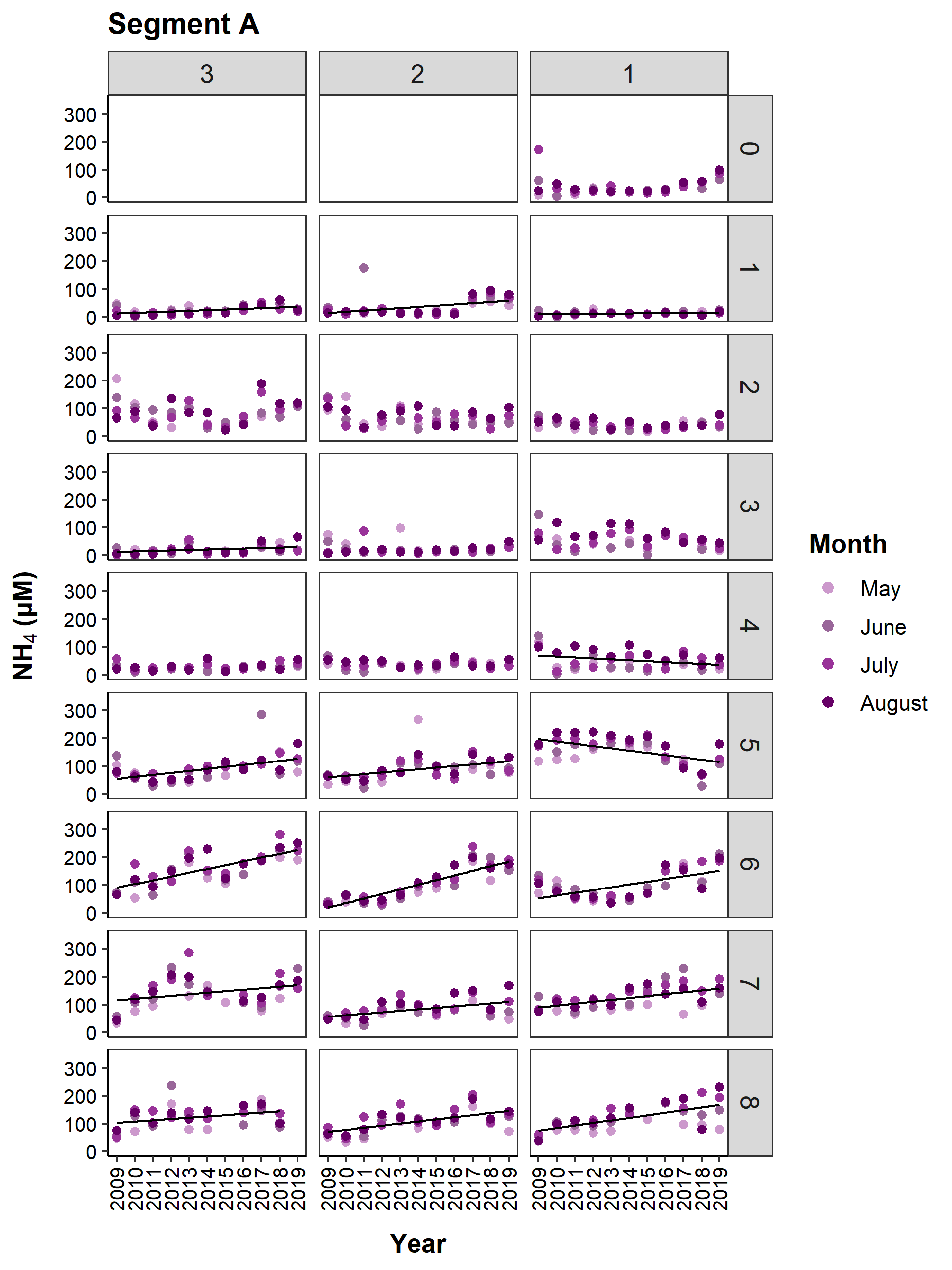

Supplement: S7 Fig — A. Monthly average NH4 concentrations (μM) for every year of the sampling period (2009–2019) at all permanent plots in Segment A. The transects at each Segment (3, 2, 1) are indicated at the top of figure, while the individual plot numbers (0–8) are indicated at the right hand side. Permanent plots with significant linear relationships (p < 0.05) between NH4 concentration and year include a best fit line on the figure which depicts the slope of the regression. (TIF) [file pone.0278215.s008.tif]

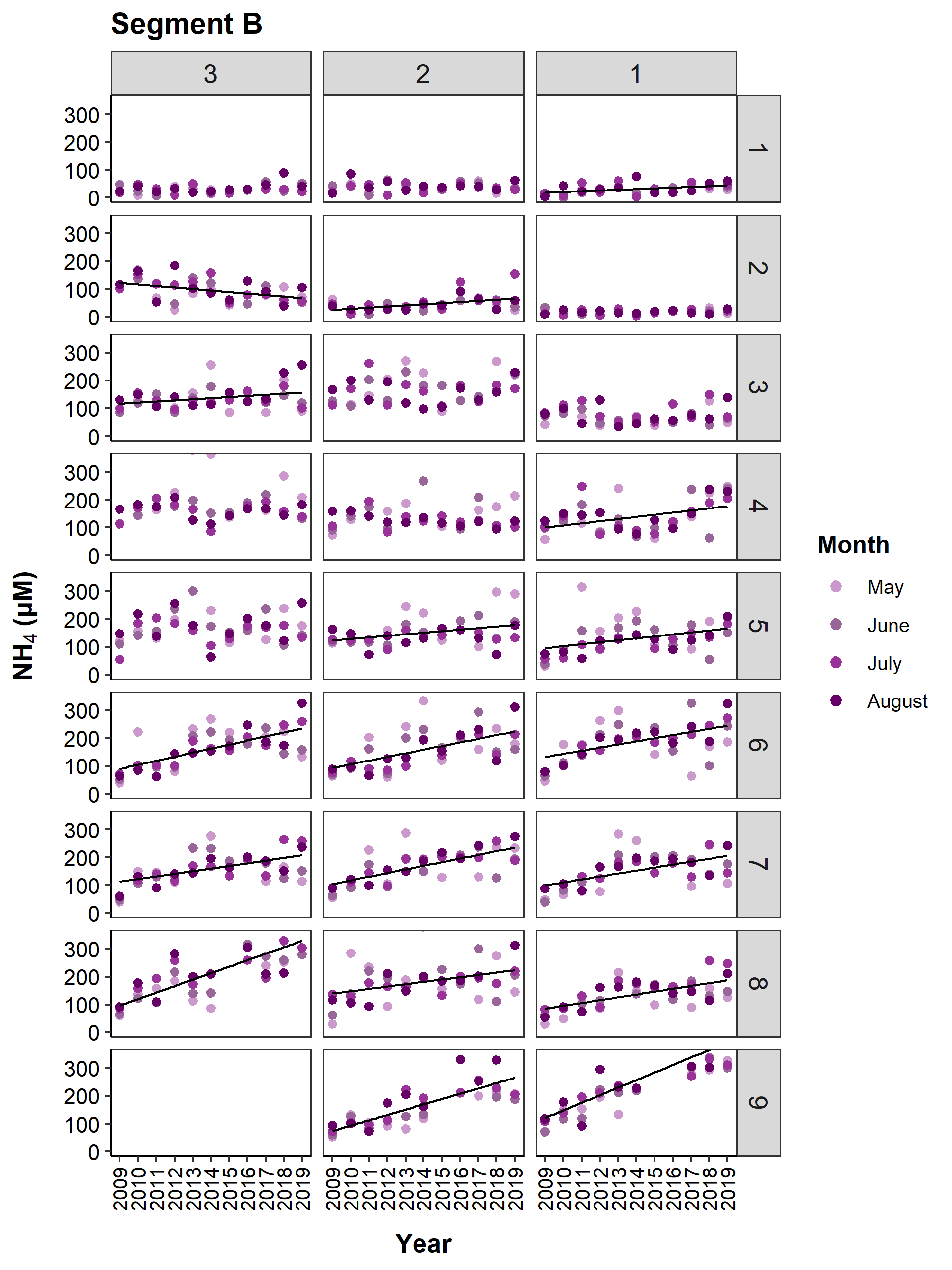

Supplement: S8 Fig — Monthly average NH4 concentrations (μM) for every year of the sampling period (2009–2019) at all permanent plots in Segment B. The transects at each Segment (3, 2, 1) are indicated at the top of figure, while the individual plot numbers (1–9) are indicated at the right hand side. Permanent plots with significant linear relationships (p < 0.05) between NH4 concentration and year include a best fit line on the figure which depicts the slope of the regression. (TIF) [file pone.0278215.s009.tif]

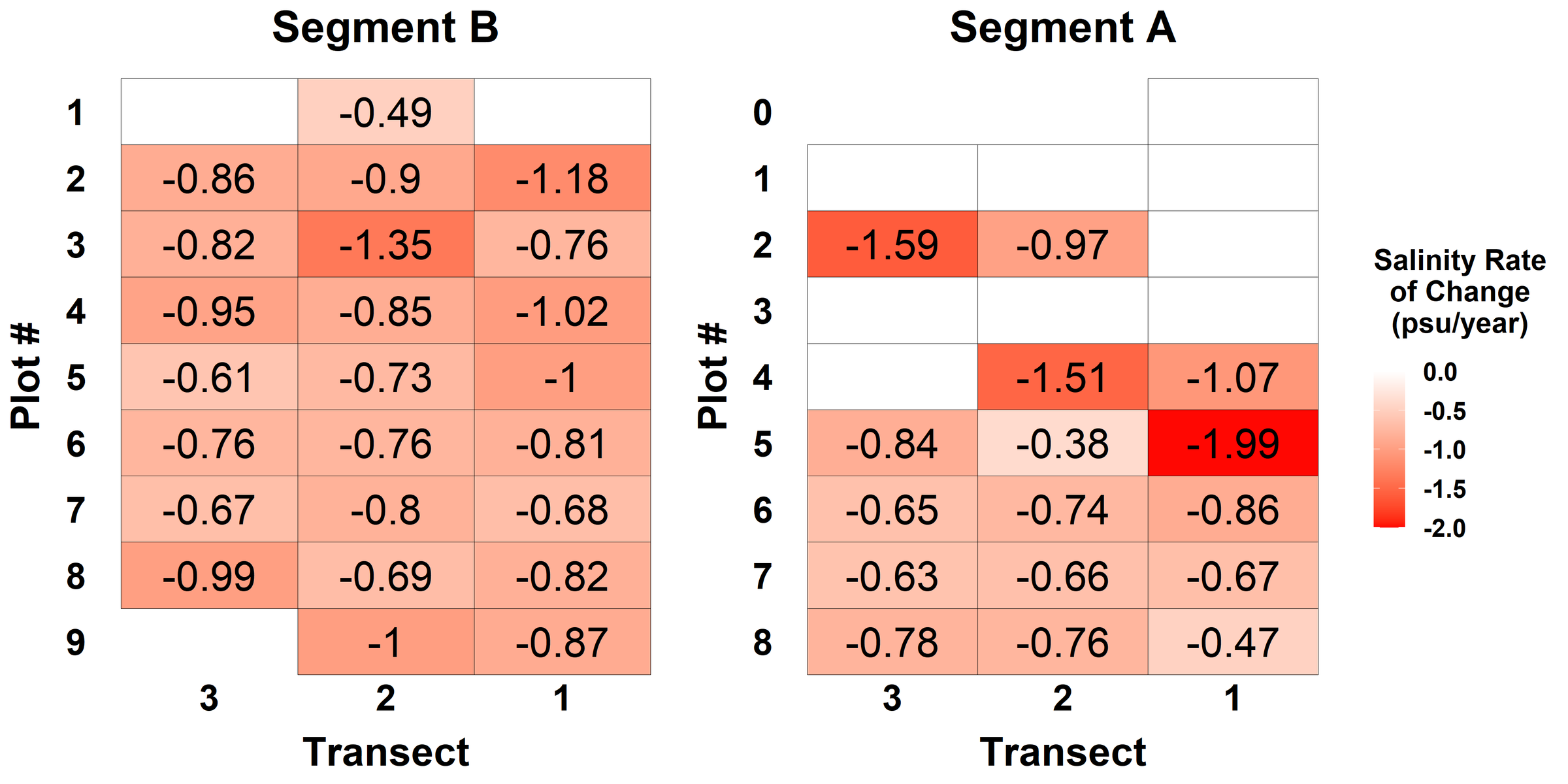

Supplement: S9 Fig — Rates of change in salinity (psu) corresponding to each permanent vegetation monitoring plot across Segments A and B. The numbers in each plot represent the slope of the linear fit between average monthly porewater salinities (from May-August) and years from 2009–2019 where p < 0.05. Plot shading visualizes the spatial variability in rates of change, and plots with no number/shading had non-significant temporal trends. Segment B is upstream and west of Segment A, so they are plotted here as they are oriented in the field. (TIF) [file pone.0278215.s010.tif]

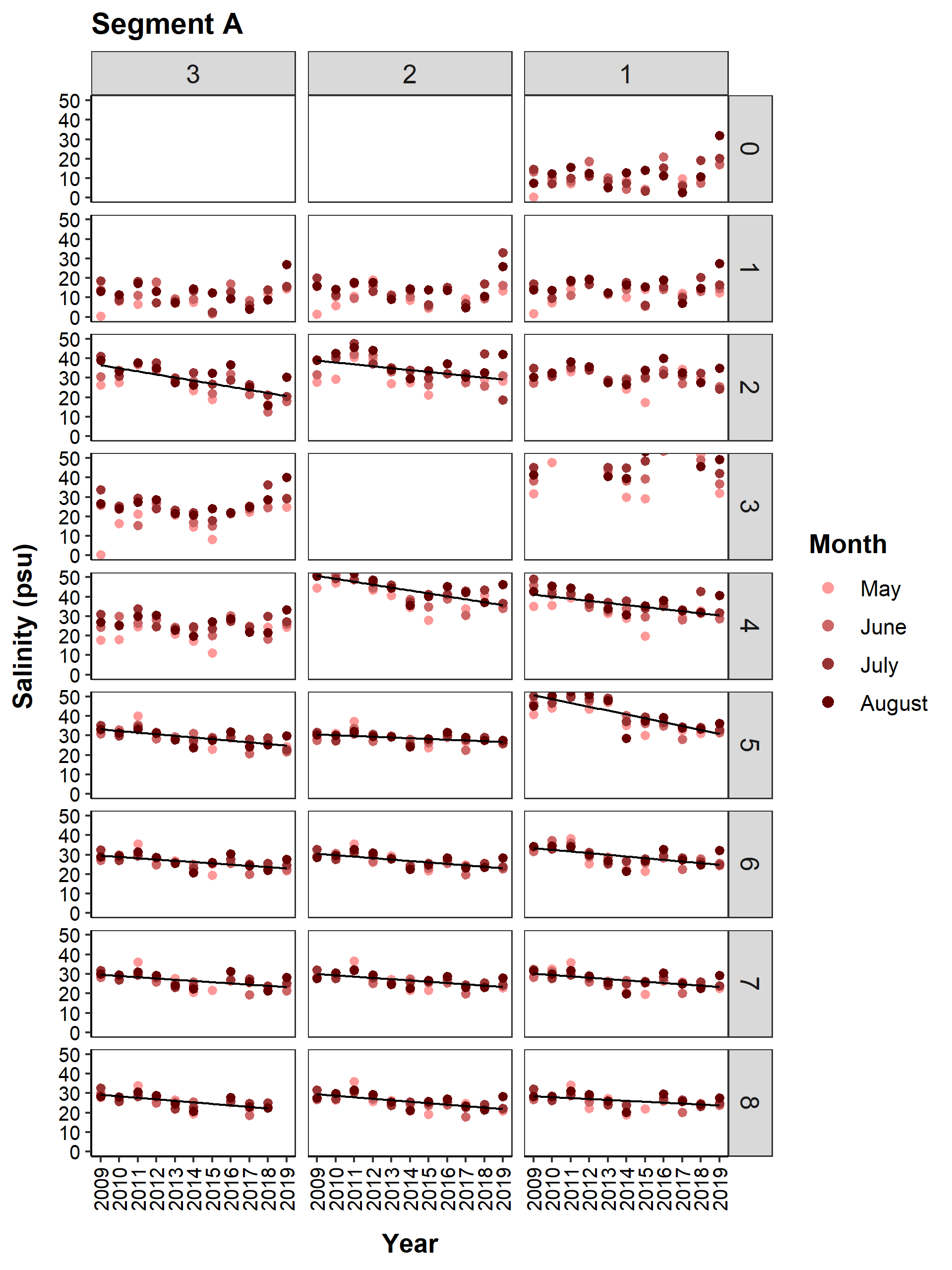

Supplement: S10 Fig — Monthly average salinities (psu) for every year of the sampling period (2009–2019) at all permanent plots in Segment A. The transects at each Segment (3, 2, 1) are indicated at the top of figure, while the individual plot numbers (0–8) are indicated at the right hand side. Permanent plots with significant linear relationships (p < 0.05) between salinity and year include a best fit line on the figure which depicts the slope of the regression. (TIF) [file pone.0278215.s011.tif]

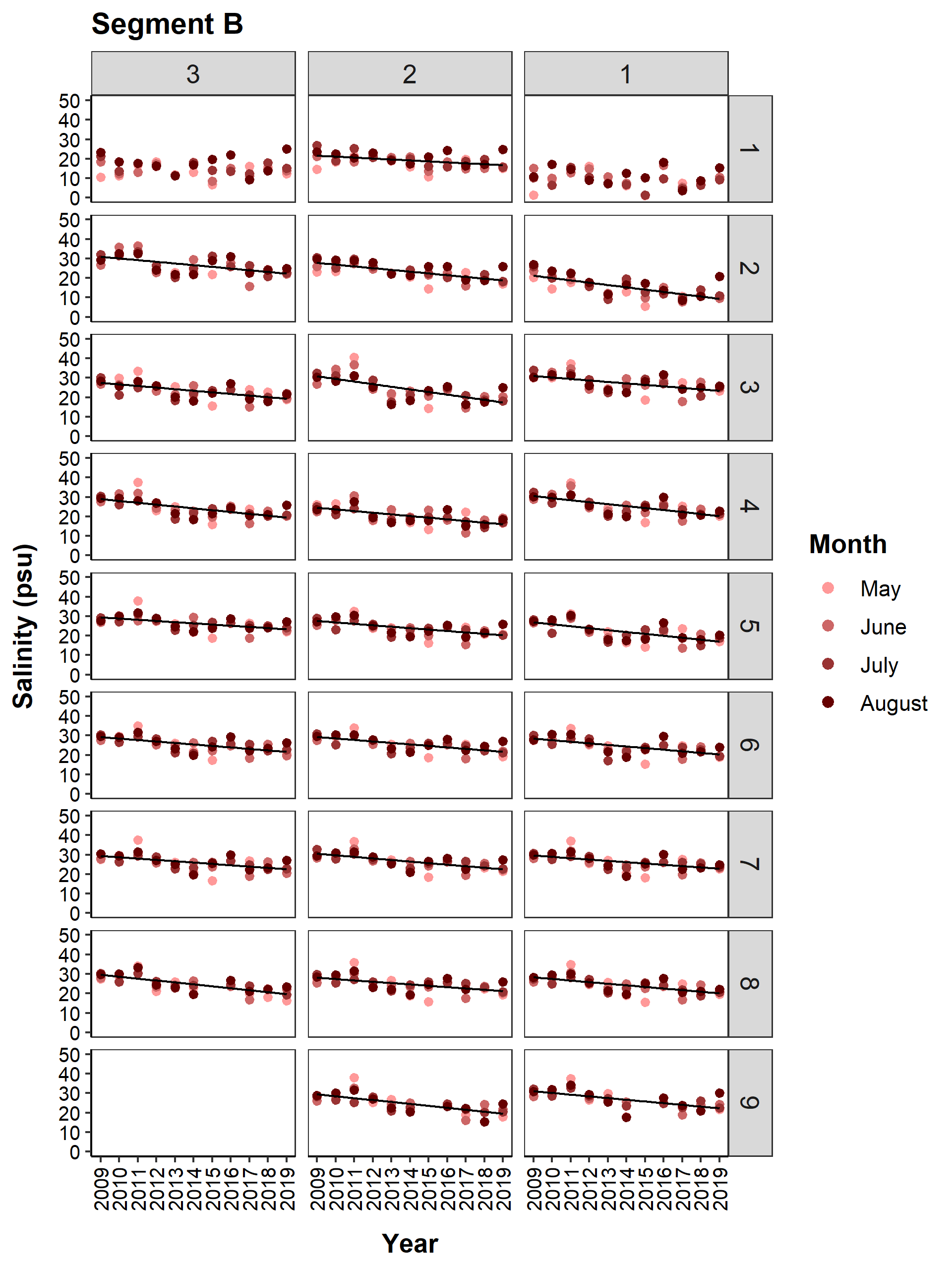

Supplement: S11 Fig — Monthly average salinities (psu) for every year of the sampling period (2009–2019) at all permanent plots in Segment B. The transects at each Segment (3, 2, 1) are indicated at the top of figure, while the individual plot numbers (1–9) are indicated at the right hand side. Permanent plots with significant linear relationships (p < 0.05) between salinity and year include a best fit line on the figure which depicts the slope of the regression. (TIF) [file pone.0278215.s012.tif]

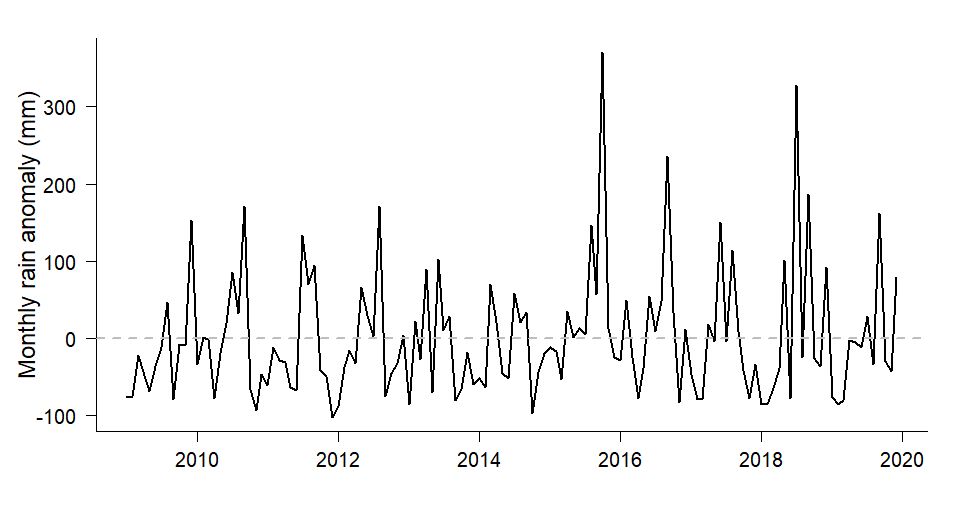

Supplement: S12 Fig — The anomaly corresponds to deviation from the long-term mean monthly precipitation from 2009–2019 (gray line). A linear regression in which the predictor was date and the response was precipitation anomaly revealed no significant trend (F 1,130 = 1.792, p = 0.183). The highest spikes in the second half of the time series correspond to large tropical storm/hurricane events that took place after the summer vegetation growing season which is when porewater nutrients were measured. (TIF) [file pone.0278215.s013.tif]
